# Leveraging Spot–Gene Heterogeneous Graphs for Unified Spatially Resolved Transcriptomics Domain Detection on Single-Slice and Multi-Slice Data

**DOI:** 10.3390/genes17030310

**Published:** 2026-03-07

**Authors:** Lina Xia, Zhenyue Ding, Xun Zhang, Kun Qian, Hongwei Li

**Affiliations:** 1 School of Mathematics and Physics, China University of Geosciences, Wuhan 430074, China; xln@cug.edu.cn (L.X.); dingyusheng064@gmail.com (Z.D.); xunnzz@163.com (X.Z.); 2 School of Mathematical Sciences and Center for Statistical Science, Peking University, Beijing 100871, China; kunqian@stu.pku.edu.cn

**Keywords:** spatially resolved transcriptomics, single-slice and multi-slice, domain detection, spot–gene heterogeneous graph, contrastive learning

## Abstract

**Background**: Spatially resolved transcriptomics (SRT) enables simultaneous measurement of gene expression and spatial location, but the existing domain detection methods are limited by over-reliance on spot-to-spot proximity, rigid pre-alignment requirements for multi-slice datasets, and inadequate mitigation of batch effects. This study aims to develop a unified method for accurate spatial domain identification across both single-slice and multi-slice SRT datasets. **Methods**: We propose a novel method named spatially resolved transcriptomics heterogeneous graph contrastive learning (stHGCL), which integrates a spot–gene heterogeneous graph, a dual-stage encoder (comprising LightGCN and GCN), and a neighborhood-driven contrastive learning module. The heterogeneous graph captures high-order structural information through spot–gene connections mediated by shared genes; the dual-stage encoder refines spot embeddings by fusing gene expression and spatial location; contrastive learning enhances intra-cluster compactness and mitigates batch effects. **Results**: stHGCL was validated on seven benchmark datasets from platforms including 10x Visium, BaristaSeq, STARmapSeq, Slide-seq, and Stereo-seq. It outperformed nine single-slice and eight multi-slice state-of-the-art methods. It achieved the highest mean Adjusted Rand Index (ARI) and Normalized Mutual Information (NMI) scores and could accurately delineate complex spatial domains with distinct boundaries, and even achieved cross-slice spatial domain detection for unaligned multi-slice datasets. Ablation studies confirmed the effectiveness of its main modules. **Conclusions**: stHGCL effectively captures high-order structural and spatial information and mitigates batch effects. It provides a robust scalable solution for unified spatial domain detection in SRT, facilitating insights into the spatial domains across both single-slice and multi-slice experimental paradigms.

## 1. Introduction

Spatially resolved transcriptomics (SRT) has come to be established as a transformative technology, gaining significant prominence for its unique capacity to measure the spatial location of spots while maintaining gene expression profiles [1,2,3]. Through diverse modalities like 10x Visium [4], BaristaSeq [5], STARmapSeq [6], Slide-seq [7], and Stereo-seq [8], researchers can now retrieve high-dimensional transcriptomic data while strictly maintaining the original physical coordinates of each capture unit. Among the core functionalities of SRT data lie the detection and delineation of spatial domains [9,10], entities that are defined as aggregates of cells or spots with comparable gene expression profiles. By offering a holistic perspective on spatial organization, such techniques collectively allow the detection and delineation of spatial domains, thus enhancing our comprehension of spatial domains and intercellular crosstalk in intricate biological contexts [11].

In the analytical pipeline for spatially resolved transcriptomics, the assignment of captured spots to their corresponding spatial domains via unsupervised clustering constitutes an essential step in biological investigations. Non-spatial analytical methods rely exclusively on gene expression profiles for cluster analysis. A typical example is Scanpy [12] (version 1.9.6), a widely used open-source toolkit for single-cell and spatial transcriptomics data analysis that only processes gene expression profiles without incorporating spatial location information. To address this limitation, a series of spatial-aware methods have been developed specifically for single-slice SRT datasets. One pioneering representative is SpaGCN [13], which integrates multi-modal data for SRT clustering by leveraging graph convolutional networks (GCNs) to fuse gene expression profiles, spatial coordinate information, and histological image datasets, thus laying the foundation for delineating multi-modal spatial domains. Following this, STAGATE [14], which adopts a graph attention autoencoder-based architecture, enhances the model’s ability to capture subtle spatial-gene relationships by assigning adaptive weights to neighboring spots, a design that improves the model’s sensitivity to local spatial variations. Going a step further, stLearn [15] expands the integration scope to include histological images, utilizing histological image features to not only improve clustering accuracy but also enable the inference of spot types and spatial trajectories. In parallel with these multi-modal strategies, another category of methods focuses on advanced graph learning methods: CCST [16], SpaceFlow [17], and GraphST [18] all employ graph contrastive learning (e.g., deep graph infomax and DGI) to refine the representation learning of capture spots, emphasizing the discriminative power of spatial adjacency. Complementing this, SEDR [19] and DeepST [20] utilize graph variational autoencoders, which prioritize learning stable embedding representations to enhance the robustness of domain identification against technical noise.

As spatially resolved transcriptomics research advances toward complex cohorts, multi-slice dataset analysis has become increasingly prevalent. Treating each slice in isolation obscures meaningful cross-sample comparisons, making the simultaneous detection of spatial domains across multi-slice datasets a critical requirement for accurate biological interpretation. In the non-spatial realm, methods like Harmony [21] have proven to be effective for batch correction in single-cell expression profiles and can be extended to cluster identification across multiple SRT slices, but their failure to incorporate spatial context limits their ability to preserve spatial domains. To address this challenge, numerous spatially explicit methodologies have been refined to underpin multi-slice analytical workflows. As a case in point, STAGATE and GraphST extend their original single-slice architectures to accommodate multi-slice datasets, yet their applicability is constrained by the prerequisite of pre-aligned spatial coordinates—a constraint that is frequently impractical in real-world research scenarios. In contrast, stLearn, DeepST and SEDR exhibit superior flexibility in spatial domain delineation for both aligned and unaligned slices, thereby eliminating the need for stringent preprocessing protocols. As an extension of STAGATE, STAligner [22] further advances unaligned slice integration by leveraging mutual nearest neighbors in the latent space, although it remains sensitive to noise in low-quality datasets. In recent research efforts, spCLUE [23] has been engineered as a comprehensive analytical framework that facilitates integrated analysis of both single- and multi-slice datasets via the synergistic integration of a multi-view graph network and contrastive learning methodologies.

In this study, we present stHGCL (spatially resolved transcriptomics heterogeneous graph contrastive learning), a unified analytical method tailored for the analysis of SRT datasets that covers both single-slice and multi-slice datasets. By integrating a dual-stage heterogeneous graph encoder with a contrastive learning method, stHGCL generates low-dimensional spot and gene embeddings through its dual-stage architecture, thereby addressing a series of critical challenges pertaining to domain annotation in SRT studies. First, the existing methods predominantly rely on spot–spot proximity for spatial modeling, which not only restricts the capture of indirect inter-slice relationships but also imposes rigid pre-alignment requirements for multi-slice data (e.g., STAGATE and GraphST) that are rarely feasible in practical experiments. Drawing inspiration from the proven efficacy of heterogeneous graph learning methodologies in single-cell analysis [24,25], stHGCL introduces an innovative spot–gene bipartite graph framework. By linking spots from different slices to a common set of gene nodes, our model enables indirect inter-slice connectivity via co-expressed gene pairs, effectively bypassing the need for physical coordinate alignment while preserving biological consistency. Second, most state-of-the-art methods adopt homogeneous graph structures that either overlook high-order spot–gene interactions or fail to adequately aggregate the local spatial context. For instance, SpaGCN focuses on multi-modal integration but lacks explicit modeling of hierarchical spot–gene relationships, while SEDR prioritizes stable embeddings but neglects fine-grained spatial aggregation. The core of stHGCL lies in its dual-stage encoder system, comprising a LightGCN encoder and a GCN encoder. This unique configuration allows the model to first learn high-order gene–spot relationships through collaborative filtering and subsequently refine spot representations by aggregating the local spatial context, capturing structural information that homogeneous methods often miss. Furthermore, standard contrastive learning approaches for SRT are typically instance-level, failing to leverage the inherent spatial neighborhood structure of spots and struggling to mitigate batch effects in multi-slice integration (e.g., Harmony ignores spatial context, while STAligner is sensitive to noise in low-quality data). We integrate a contrastive learning module specifically tailored for SRT data: unlike other methods, stHGCL employs a neighborhood-based constraint to ensure intra-cluster compactness while leveraging the heterogeneous structure to mutually supervise and correct representations across slices, effectively mitigating batch effects. Finally, the architectural merits of stHGCL against alternative methods are summarized in Table S1. To guarantee a rigorous evaluation, we validated our analytical framework by leveraging seven representative benchmark datasets spanning a diverse range of technical platforms, namely 10x Visium, BaristaSeq, STARmapSeq, Slide-seqV2 and Stereo-seq. Across both single-slice and multi-slice experimental datasets, stHGCL demonstrates robust spatial clustering capability and high precision in domain demarcation, outperforming prevailing state-of-the-art methodologies in terms of both analytical accuracy and computational efficacy.

## 2. Materials and Methods

### 2.1. Overview of stHGCL

stHGCL acts as a unified method for detecting spatial domains in both single-slice and multi-slice SRT datasets. To efficiently fuse gene expression and spatial localization information, it comprises a spot–gene heterogeneous graph, a dual-stage encoder system, and a neighborhood-driven contrastive learning module. The unified mathematical model is formulated as follows.

With respect to a specified SRT dataset comprising *B* slices, each slice possesses both a gene expression matrix and its corresponding spatial location. The dataset is denoted as $D=\left\{X_{1},\ldots ,X_{B}\right\}\cup \left\{U^{1},\ldots ,U^{B}\right\}$, where $X_{b}\in R^{N_{b}\times G}$ and $U^{b}\in R^{N_{b}\times 2}$ represent the gene expression matrix and corresponding spatial location of slice *b* (b=1,…,B), respectively. In the current framework, $N_{b}$ and *G* represent the numbers of spots and genes within the *b*-th slice. With respect to this slice, $x_{i}^{\left(b\right)}$ is designated as the gene expression vector corresponding to the *i*-th spot, and $u_{i}^{\left(b\right)}$ refers to its corresponding spatial coordinate information. stHGCL aims to develop a joint mapping function $f:R^{G}\times R^{2}\to R^{d}$ (d≪G), which compresses high-dimensional spot information into a low-dimensional latent embedding. Subsequently, the latent spot embeddings are derived by

$$Z=\left\{z_{i}^{\left(b\right)}|z_{i}^{\left(b\right)}=f\left(x_{i}^{\left(b\right)},u_{i}^{\left(b\right)}\right);\;\;i=1,2,\ldots ,N_{b},\;\;b=1,2,\ldots ,B\right\},$$

where $z_{i}^{\left(b\right)}$ represents the embedding associated with the *i*-th spot in the *b*-th slice. For spatial domain detection, the task is defined as finding a partition:

$$T=\left\{C_{m}|m=1,2,\ldots ,K\right\},\;s.t.\;Z=\overset{K}{\underset{m=1}{⋃}}C_{m},\;C_{m}\cap C_{n}=⌀\;\left(m\neq n\right),$$

where *K* is the number of clusters, $C_{m}$ denotes the *m*-th cluster, and T represents the set of all clusters.

**Figure 1:**
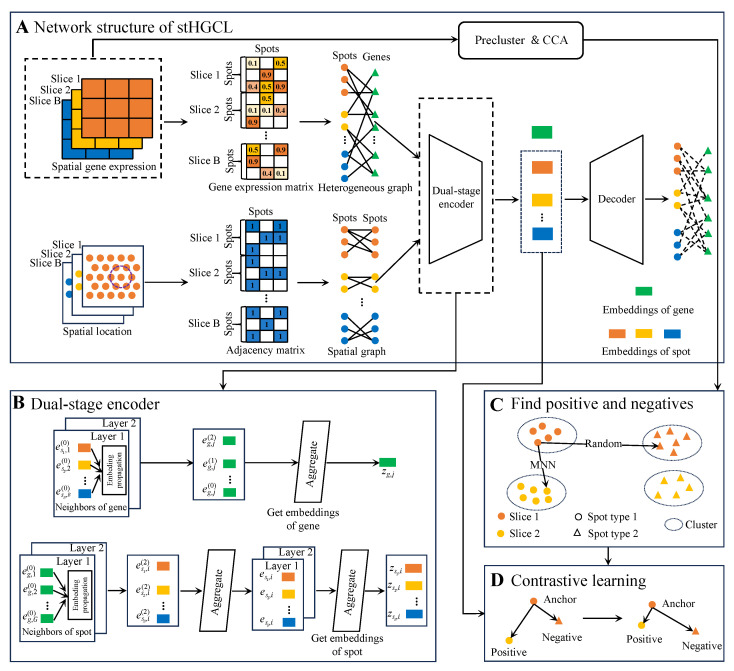
stHGCL workflow overview. (**A**) As illustrated, the stHGCL framework takes gene expression profiles and spatial locations across multiple slices as key analytical inputs. This architecture constructs a spot–gene heterogeneous graph and a spatial adjacency matrix to model biological relationships. Consequently, the model yields spatial spot embeddings for spatial domain detection. (**B**) The model captures both spot and gene node embeddings by integrating transcriptional profiles with spatial locations. (**C**) The construction of anchor–positive and anchor–negative pairs. (**D**) The contrastive learning module is designed to impose restrictions on the spot node embeddings output by the encoder such that anchor–positive pairs present mutual attraction whereas anchor–negative pairs manifest mutual repulsion.

For multi-slice analysis, stHGCL constructs a comprehensive heterogeneous graph by connecting spots from all experimental slices to a shared set of gene nodes. This architecture facilitates indirect inter-slice connectivity through co-expressed genes, bypassing the need for rigid physical coordinate alignment. The workflow proceeds through three primary stages (Figure 1). (A) Heterogeneous graph construction: Interaction relationships between spots and genes are utilized as edges to capture high-order structural information across different slices. (B) Dual-stage encoding: The model employs a dual-stage encoder, which is a multi-layer heterogeneous graph neural network (HetGNN) leveraging LightGCN [26]. It preliminarily learns spot embeddings and final gene embeddings. A GCN encoder follows, which aggregates local spatial locations from a spot-neighborhood graph to refine the final spot embeddings. (C) and (D) contrastive learning: To eliminate batch effects and ensure intra-class compactness, a contrastive learning module constrains spot embeddings. It employs mutual nearest neighbors (MNNs) in the canonical correlation analysis (CCA) space as inter-slice anchor–positive pairs while selecting anchor–negative pairs from distinct clusters generated via the Louvain algorithm [27] to maintain biological consistency across slices. For the single-slice analysis (B=1), the model retains the heterogeneous graph and dual-stage encoder but adopts a simplified contrastive learning module. Specifically, for single-slice analysis (B=1), inter-slice MNN pairing is omitted. Anchor–positive pairs are solely defined by intra-slice k-nearest neighbors based on Euclidean distances in the preprocessed expression matrix. Anchor–negative pairs are randomly selected from distinct pre-clusters generated by the Louvain algorithm, ensuring consistency with the core contrastive learning logic while simplifying multi-slice-specific steps.

In the following sections, we first elaborate on the constituent elements of the stHGCL framework for the case of multi-slice datasets (B>1) and then demonstrate how the model may be tailored to single-slice data (B=1).

### 2.2. Multi-Slice Spatially Resolved Transcriptomics Domain Detection (B>1)

For multi-slice SRT domain detection, we take multi-slice SRT matrices ($X_{b}\in R^{N_{b}\times G}$ and $U^{b}\in R^{N_{b}\times 2}$) as inputs, where $N_{b}\;\left(b=1,2,...,B\right)$ denotes the number of spots in slice *b*; *G* denotes the number of genes; *B* represents the total number of slices for multi-slice data. The stHGCL method for multi-slice data spatial domain detection contains the following main steps: (1) data preprocessing; (2) construction of the spot–gene heterogeneous graph; (3) graph encoder and decoder used to extract information; (4) contrastive learning; (5) stHGCL self-supervised training loss. The specific functions of each step are elaborated below.

#### 2.2.1. Data Preprocessing

Suppose that X represents a spatial gene expression dataset containing *B* slices:

$${\tilde{X}}_{b}=\left[{\tilde{x}}_{ij}^{b}\right]\in R^{N_{b}\times G^{0}},$$

where $N_{b}$ denotes the number of spots in slice *b*, $G^{0}$ represents the initial number of genes, and ${\tilde{X}}_{b}$ signifies the initial count matrix of slice *b* containing $N_{b}$ spots and $G^{0}$ genes.

Initially, raw gene expression counts are filtered using the SCANPY package [12]. To focus on the most informative biological signals, we filter for the 2000 highly variable genes (HVGs) using Scanpy’s highly_variable_genes function with the seurat flavor [12]. Following this, the dimensionality of this feature space is compressed via principal component analysis (PCA), resulting in a 200-dimensional embedding that acts as the primary input for stHGCL method, and the filtered spot expression matrix is denoted as $X_{b}=\left(x_{ij}^{b}\right)\in R^{N\times G}$, where $x_{ij}^{b}$ represents the expression value of the gene *j* in the spot *i*, *N* is the number of spots, and *G* is the number of genes. The library size factor for each spot is then calculated as

(1) $$l_{i}^{b}=\frac{\sum_{j=1}^{N}{\tilde{x}}_{ij}^{b}}{{\mathrm{Median}}_{i^{\prime }}\left\{\sum_{j=1}^{N}{\tilde{x}}_{i^{\prime }j}^{b}\right\}}.$$

Next, the counts are normalized using the library size factor and log-transformed to obtain the standardized expression values:

(2) $$x_{ij}^{b}=\log\left(\frac{{\tilde{x}}_{ij}^{b}}{l_{i}^{b}}+1\right).$$

The normalized expression value is denoted as $x_{ij}^{b}\in R^{N_{b}\times G}$. The processed expression matrix is denoted as $X=\{X_{b}\in R^{N_{b}\times G}|b=1,2,...,B\}$ and will be used as input to stHGCL.

To capture the spatial architecture of multiple slices, we construct a spatial adjacency graph for each slice *b*, denoted as $G_{A}^{b}\left(U^{b},E_{A}^{b}\right)$. Here, $U^{b}=\left\{u_{1}^{b},u_{2}^{b},\ldots ,u_{N_{b}}^{b}\right\}$ signifies the collection of $N_{b}$ spots, and $E_{A}^{b}$ constitutes the edges reflecting their physical proximity. We determine the connectivity by calculating the Euclidean distance between spot locations. For each spot, its local neighborhood is defined by its *k*-nearest neighbors. Following numerical experiments across diverse SRT datasets, we found that a constant value of k=7 results in optimal model performance. This neighbor selection process is characterized as

$$D_{i}^{b}=\left\{\sqrt{{\left(u_{i,x}^{b}-u_{I,x}^{b}\right)}^{2}+{\left(u_{i,y}^{b}-u_{I,y}^{b}\right)}^{2}}\;|\;I\in \left\{1,2,\ldots ,N_{b}\right\},I\neq i\right\},$$

$$N_{i}^{b}=\mathrm{Sort}\left(D_{i}^{b},k\right),$$

where $D_{i}^{b}$ comprises the distance values between spot *i* and the remaining spots within the *b* slice, $u_{i}^{b}=\left(u_{i,x}^{b},u_{i,y}^{b}\right)$ denotes the spatial coordinates of the *i*-th spot in the *b* slice, $N_{i}^{b}$ represents the top *k*-nearest neighbors assigned to spot *i* in the *b* slice, and Sort(·) denotes the sort and select operation.

Finally, the multi-slice spatial graph $G_{A}=\left\{G_{A}^{b}|b=1,2,\ldots ,B\right\}$ is constructed by integrating the spatial graphs of each slice.

#### 2.2.2. Construction of the Spot–Gene Heterogeneous Graph

For multi-slice SRT data, we construct a bipartite graph $G_{H}^{b}\left(U_{b},W,C_{b}\right)$ for slice *b* from the raw gene expression data $X_{b}$, effectively characterizing the link between spots and genes for every slice, where $U_{b}$ and W denote the sets of spot nodes in slice *b* and gene nodes, respectively. The adjacency matrix $C_{b}\in R^{\left(N_{b}+G\right)\times \left(N_{b}+G\right)}$ is as follows:

(3) $$C_{b}=\left(\begin{array}{cc} O_{1}^{b} & R_{b}\\ R_{b}^{T} & O_{2}^{b} \end{array}\right),$$

where $N_{b}$ and *G* denote the number of spot nodes in slice *b* and gene nodes, respectively; $O_{1}^{b}\in R^{N_{b}\times N_{b}}$ and $O_{2}^{b}\in R^{G\times G}$ are zero matrices; and $R_{b}=\left[r_{ij}^{b}\right]\in R^{N_{b}\times G}$ is the spot–gene adjacency matrix, where 1 represents a positive edge and 0 denotes a negative one:

(4) $$r_{ij}^{b}=\left\{\begin{array}{cc} 1, & \mathrm{if}\;\mathrm{gene}\;j\;\mathrm{is}\;\mathrm{expressed}\;\mathrm{in}\;\mathrm{spot}\;i\;\mathrm{of}\;\mathrm{slice}\;b,\\ 0, & \mathrm{otherwise}. \end{array}\right.$$

Finally, the heterogeneous graph $G_{H}=\left\{G_{H}^{b}|b=1,2,\ldots ,B\right\}$ is constructed by integrating the bipartite graphs, thereby mapping the indirect interactions between spots from distinct experimental slices.

#### 2.2.3. A Dual-Stage Encoder and Dot Product Decoder for Heterogeneous Information Extraction

To extract structural information regarding spots and genes from the heterogeneous graph $G_{H}$ and the spatial graph $G_{A}$, stHGCL leverages a dual-stage encoder and a decoder: a LightGCN encoder to obtain preliminary spot embeddings and final gene embeddings, a GCN encoder to obtain final spot embeddings that incorporate spatial information aggregation, and a dot product decoder to reconstruct the heterogeneous graph.

In the LightGCN encoder [26], we represent each capture spot *i* (for slice *b*) and gene *j* using initial embeddings, $e_{s_{b},i}^{\left(0\right)}\in R^{d}$ and $e_{g,j}^{\left(0\right)}\in R^{d}$, respectively. These vectors are treated as learnable parameters and undergo random initialization, with *d* specifying the latent space dimensionality. Subsequently, the convolution operation for the *k*-th layer of this graph-based encoder is defined as follows:

(5) $$\left\{\begin{array}{c} \begin{array}{cc} e_{s_{b},i}^{\left(k\right)} & =\mathrm{Aggregate}\{e_{g,j}^{\left(k-1\right)}|j\in N_{s_{b},i}\}\\ \; & =\sum_{j\in N_{s_{b},i}}\frac{1}{\sqrt{|N_{s_{b},i}|}\sqrt{|N_{g,j}|}}e_{g,j}^{\left(k-1\right)},\;\;b=1,2,...,B, \end{array}\\ e_{g,j}^{\left(k\right)}=\mathrm{Aggregate}\{e_{s_{b},i}^{\left(k-1\right)}|i\in N_{g,j}\}=\\ \;\;=\frac{1}{B}\sum_{b=1}^{B}\sum_{i\in N_{g,j}}\frac{1}{\sqrt{|N_{s_{b},i}|}\sqrt{|N_{g,j}|}}e_{s_{b},i}^{\left(k-1\right)}, \end{array}\right.$$

where k∈{1,2,…,K} denotes the number of layers of the LightGCN encoder. The sets $N_{s_{b},i}$ and $N_{g,j}$ delineate the first-order neighborhood for spot *i* on slice *b* and gene *j* within the heterogeneous graph $G_{H}^{b}$, respectively. The terms $|N_{s_{b},i}|$ and $|N_{g,j}|$ reflect the degree (neighbor count) of these respective nodes. To prevent the numerical instability or uncontrolled scaling of embeddings during message passing, we employ the symmetric normalization factor $1/\sqrt{|N_{s_{b},i}|}\sqrt{|N_{g,j}|}$.

After *K* graph convolution layers, stHGCL obtains a set of K+1 embeddings for both spots and genes. Subsequently, a weighted sum of these multi-layer embeddings is calculated to alleviate the over-smoothing phenomenon. The final encoded embeddings for spot *i* in slice *b* and gene *j* are formulated as

(6) $$\left\{\begin{array}{c} e_{s_{b},i}=\sum_{k=0}^{K}w^{\left(k\right)}e_{s_{b},i}^{\left(k\right)},\;\;b=1,2,...,B,\\ z_{g,j}=\sum_{k=0}^{K}w^{\left(k\right)}e_{g,j}^{\left(k\right)}, \end{array}\right.$$

where $w^{\left(k\right)}$ are the weights for each layer, ranging from 0 to 1. In our implementation, we set K=2 and assign uniform weights $w^{\left(k\right)}=1/\left(K+1\right)$ to achieve optimal performance. Following a two-layer LightGCN encoder, $e_{s_{b},i}$ is the *i*-th preliminary spot embedding for slice *b* (without spatial information), and $Z_{g}={\left\{z_{g,j}\right\}}_{j=1}^{G}$ is the final gene embedding.

Following the LightGCN encoder, we obtain the preliminary spot embeddings that incorporate similar feature information. Given that spots in spatially resolved transcriptomics are more likely to belong to the same domain type if they are spatially proximate, we perform an additional round of spatial information aggregation via GCN, leveraging the previously constructed undirected neighborhood graph. The embedding of spot *i* in slice *b* at the *v* th layer is updated as follows:

(7) $$z_{s_{b},i}^{\left(v\right)}=\sum_{h\in E_{A}^{b}\left(i\right)}\frac{1}{|E_{A}^{b}\left(i\right)|}z_{s_{b},h}^{\left(v-1\right)},\;\mathrm{with}\;z_{s_{b},i}^{\left(0\right)}=e_{s_{b},i},$$

where $z_{s_{b},i}^{\left(v\right)}$ denotes the embedding of spot *i* in slice *b* after passing through the *v*-th aggregation layer via the neighborhood graph, $E_{A}^{b}\left(i\right)$ refers to the neighbor set of spot *i* within this graph, and *h* signifies the *h*-th spot contained in the neighbor set corresponding to spot *i*. Upon completing two layers of spatial propagation, the final spot embeddings for slice *b* are denoted as $Z_{s_{b}}={\left\{z_{s_{b},i}^{\left(2\right)}\right\}}_{i=1}^{N_{b}}$.

In order to reduce the risk of biological information loss throughout the encoding process, a dot product decoder is utilized to reconstruct the heterogeneous graph. This method ensures that the structural integrity of the data is preserved within the learned embeddings. Specifically, the reconstructed feature matrix ${\hat{X}}_{b}$ is generated by calculating the proximity between the final gene embeddings $Z_{g}$ and spot embeddings $Z_{s_{b}}$ as follows:

(8) $${\hat{X}}_{b}=\sigma \left(Z_{g}Z_{s_{b}}^{T}\right),$$

where σ stands for the sigmoid activation function.

#### 2.2.4. Contrastive Learning

To enhance the performance of spatial transcriptomic data integration, minimize redundant inter-spot information, and generate spot embeddings with enhanced discriminative power, a contrastive learning module is integrated to preserve the neighborhood structural information of spots.

For the inter-slice similarity structure between spots, the sampling method of anchor–positive pairs and anchor–negative pairs in triplets is critical for the effectiveness of the contrastive learning. CCA is utilized to capture correlated gene modules between two datasets and preserve the correlations among them [28]. With respect to any two distinct slices $b_{1}$ and $b_{2}$, CCA is employed to reduce the dimensionality of the preprocessed expression matrices $X_{b_{1}}\in R^{N_{b_{1}}\times G}$ and $X_{b_{2}}\in R^{N_{b_{2}}\times G}$. First, the correlation matrix $Y\in R^{N_{b_{1}}\times N_{b_{2}}}$ representing the cross-slice spot similarity is computed as

$$Y=X_{b_{1}}X_{b_{2}}^{T}.$$

Subsequently, randomized Singular Value Decomposition is carried out on the correlation matrix Y:

$$Y\approx U_{1}\Sigma U_{2}^{T},$$

where $U_{1}\in R^{N_{b_{1}}\times d}$ and $U_{2}\in R^{N_{b_{2}}\times d}$ denote the dimensionality-reduced embeddings of spots from $b_{1}$ and slice $b_{2}$, respectively, and $\Sigma \in R^{d\times d}$ is a diagonal matrix with *d* representing the dimensionality after reduction. Following the initial feature extraction, we apply $L_{2}$ normalization to $U_{1}$ and $U_{2}$ to ensure scale consistency. Anchor–positive pairs are subsequently established by identifying MNNs between these subsets, guided by the neighborhood parameter $k_{\mathrm{mnn}}$. Within each multi-slice dataset, pairwise MNNs are identified between all slice pairs, and the slice with the highest number of spots is selected as the group’s representative to minimize information loss. Finally, MNNs are computed between representative datasets of different groups to ensure global cross-slice alignment.

For the intra-slice similarity structure between spots, contrastive learning encourages similar spot pairs to pull together while pushing dissimilar pairs apart. Using each capture spot as an anchor, we define positive samples by identifying its $k_{\mathrm{knn}}$ nearest neighbors according to Euclidean distances in the preprocessed matrix $X_{b}$. The hyperparameter $k_{\mathrm{knn}}$ specifies the neighborhood size. Correspondingly, for every anchor, an equal number of negative samples ($k_{\mathrm{knn}}$) are stochastically drawn from distinct clusters. These pre-clusters are preliminarily determined by utilizing the Louvain algorithm [27] on the same slice with a preset resolution *r*.

Let $T=\{\left(\tilde{a},\;\tilde{p},\;\tilde{n}\right)\}$ represent the set of triplet instances. Its members, the constituent elements $\tilde{a},\;\tilde{p}$, and $\tilde{n}$, indicate the index values associated with the anchor spot as well as its paired positive and negative samples. The contrastive learning loss $L_{\mathrm{CL}}$ is designed to ensure that the distance between positive pairs is smaller than that of negative pairs by at least a margin *l*. It is defined as follows:

$$L_{\mathrm{CL}}=\sum_{(\tilde{a},\;\tilde{p},\;\tilde{n})\in T}\max\{∥z_{s,\tilde{a}}-z_{s,\tilde{p}}{∥}_{2}-∥z_{s,\tilde{a}}-z_{s,\tilde{n}}{∥}_{2}+l,0\},$$

where $z_{s,\tilde{a}},\;z_{s,\tilde{p}}$ and $z_{s,\tilde{n}}$ denote the spot embeddings of the anchor, positive and negative obtained from the dual-stage (LightGCN and GCN) encoder, respectively; the hyperparameter *l* stands for the margin distance between anchor–positive and anchor–negative pairs.

#### 2.2.5. stHGCL Self-Supervised Training Loss

To facilitate edge reconstruction, stHGCL stochastically samples 10% of the observed edges from the heterogeneous graph $G_{b}$ to form a positive heterogeneous subgraph $G_{H+}^{b}$, while an equivalent number of non-existent edges are selected to build a negative heterogeneous subgraph $G_{H-}^{b}$. These dual subgraphs then serve as inputs for the decoder. The reconstruction fidelity is quantified using a mean squared error loss ($L_{\mathrm{MSE}}$), calculated between the original and reconstructed count matrices:

(9) $$L_{\mathrm{MSE}}=\sum_{b=1}^{B}L_{\mathrm{MSE}}^{b},$$

where

(10) $$L_{\mathrm{MSE}}^{b}=\frac{1}{N_{b}^{+}}\sum_{\left(i,j\right)\in G_{H+}^{b}}{\left(x_{ij}^{b}-{\hat{x}}_{ij}^{b}\right)}^{2}+\frac{1}{N_{b}^{-}}\sum_{\left(i,j\right)\in G_{H-}^{b}}{\left(x_{ij}^{b}-{\hat{x}}_{ij}^{b}\right)}^{2},$$

in which b=1,2,…,B. The original and reconstructed counts of gene *j* in spot *i* for slice *b* are indicated by $x_{ij}^{b}$ and ${\hat{x}}_{ij}^{b}$. The edge counts for the heterogeneous subgraphs $G_{H+}^{b}$ and $G_{H-}^{b}$ are denoted as $N_{b}^{+}$ and $N_{b}^{-}$, respectively.

Furthermore, a regularization loss $L_{\mathrm{reg}}$ term is employed to constrain the initial spot and gene embeddings, thereby mitigating overfitting during the training process:

(11) $$L_{\mathrm{reg}}=\frac{∥E_{s}^{0}{∥}_{F}^{2}+{∥E_{g}^{0}∥}_{F}^{2}}{2(N_{\mathrm{total}}+G)},$$

where $E_{s}^{\left(0\right)}$ and $E_{g}^{\left(0\right)}$ represent the matrices of initial embeddings for spots and genes, respectively, where $E_{s}^{\left(0\right)}=\left(e_{s_{1},1}^{\left(0\right)},\ldots ,e_{s_{B},N_{B}}^{\left(0\right)}\right)$ and $E_{g}^{\left(0\right)}=\left(e_{g,1}^{\left(0\right)},\ldots ,e_{g,G}^{\left(0\right)}\right)$. The aggregate counts of all capture spots and genes are signified by $N_{\mathrm{total}}=\sum_{b=1}^{B}N_{b}$ and *G*, while ${∥\cdot ∥}_{F}^{2}$ signifies the squared Frobenius norm used for regularization.

The total loss $L_{\mathrm{total}}$ is the sum of both losses:

(12) $$L_{\mathrm{total}}=L_{\mathrm{MSE}}+\lambda_{1}L_{\mathrm{CL}}+\lambda_{2}L_{\mathrm{reg}},$$

where $\lambda_{1}$ and $\lambda_{2}$ are hyperparameters.

### 2.3. Single-Slice Spatially Resolved Transcriptomics Domain Detection (B=1)

For single-slice analysis, the input includes a matrix $X\in R^{N\times G}$ and a coordinate matrix $U\in R^{N\times 2}$. In these matrices, *N* and *G* signify the counts of spots and genes, respectively. This dataset is employed to detect spatial domains. We present the realization of spatial domain identification tailored for single-slice data. By setting the number of slices to B=1, the procedures for data preprocessing, construction of the spot–gene heterogeneous graph, and the graph encoder and decoder remain consistent with the multi-slice framework described above. The primary distinction lies in the contrastive learning module: for single-slice analysis, we utilize only intra-slice *k*-nearest neighbors to identify anchors, positives, and negatives rather than incorporating inter-slice MNNs. The objective function for single-slice data is similarly composed of the contrastive learning loss ($L_{\mathrm{CL}}$), the mean squared error loss ($L_{\mathrm{MSE}}$), and the regularization loss ($L_{\mathrm{reg}}$).

### 2.4. Cluster

We train the stHGCL model based on the loss function in Equation (12). The Adam optimizer [29] processes the parameter updates. Specific hyperparameter settings can be found in the Supplementary Methods: hyperparameter settings of stHGCL. Finally, the model produces spot embeddings ($Z_{s}$). These spot embeddings are employed to identify spatial domains within the target slice.

When conducting single-slice analysis, the sole required components are the single-slice heterogeneous graph and neighborhood graph. As spot counts in individual slices tend to be sparse, we utilize K-means to implement reliable clustering [30]. For multi-slice spatially resolved transcriptomics data, the final embeddings are clustered using the mclust algorithm [31] to achieve spatial domain detection.

## 3. Results

In the following sections, we present assessments of the efficacy of stHGCL using seven datasets derived from a broad spectrum of spatially resolved transcriptomics platforms (see Supplementary Table S2 and Supplementary Methods: baseline and competing methods for detailed specifications). To evaluate stHGCL’s performance in single-slice tasks, we compared stHGCL against nine analytical methods: the non-spatial tool Scanpy [12] and eight spatial methods (stLearn [15], SpaGCN [13], STAGATE [14], CCST [16], DeepST [20], GraphST [18], SEDR [19], and spCLUE [23]) that take spatial heterogeneity into consideration. The technical details of these methods are provided in Supplementary Table S1 and the Supplementary Methods: baseline and competing methods. Among the nine evaluated methods, stLearn, STAGATE, DeepST, GraphST, SEDR and spCLUE are capable of directly processing multi-slice datasets. Notably, GraphST and STAGATE are restricted to multi-slice data with aligned spatial coordinates; accordingly, their performance on unaligned multi-slice datasets is not reported herein. For the spatial clustering of multi-slice data, we additionally included Harmony [21] and STAligner [22] for comparison.

### 3.1. Benchmarking stHGCL Performance on the Human DLPFC Single-Slice Dataset

**Figure 2:**
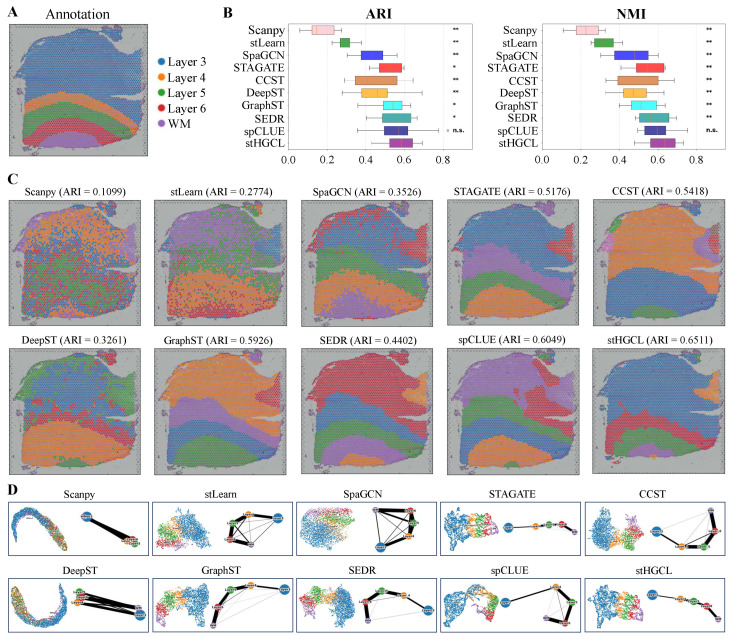
Spatial domain detection performance in the DLPFC dataset. (**A**) Annotations for slice 151669. (**B**) Distribution of ARI and NMI scores for 10 methods across 12 DLPFC slices, presented in chronological order of method development. Notations for statistical significance (stHGCL vs. other methods): n.s. *p* > 0.05; * *p* < 0.05; ** *p* < 0.005; where n.s. indicates no significant difference. (**C**) Spatial clustering visualizations produced by ten methods for slice 151669. (**D**) Integrated UMAP embedding and PAGA trajectory results for slice 151669. The thickness of edges in a PAGA graph reflects the degree of connectivity among spot groups.

In this section, we conduct a comprehensive and systematic analysis of 12 DLPFC slices captured using the 10x Visium spatially resolved transcriptomics platform. Maynard et al. [32] provided detailed annotations for each slice in the DLPFC dataset, partitioning them into four or six cortical layers and one WM layer. We selected slice 151669 as an example, which contains 3661 spatial spots and 33,538 genes. Annotation data corresponding to this 151669 slice is shown in Figure 2A.

To evaluate the clustering performance of stHGCL, we adopted the Adjusted Rand Index (ARI) [33] and Normalized Mutual Information (NMI) [34] as key evaluation metrics, both of which quantify the level of consistency between the predicted cluster assignments and the established spatial domain annotations. Based on the benchmarking results (Figure 2B), stHGCL achieved the highest mean ARI and NMI across all 12 DLPFC slices, significantly outperforming the second-ranked method spCLUE.

In summary, the outcomes yielded by the two assessment metrics confirm that stHGCL achieves superior clustering performance for the identification of spatial domains using the DLPFC dataset. ARI and NMI evaluate the agreement between predictions and annotations from different perspectives. To verify the statistical significance of the differences, we performed the Wilcoxon signed-rank test. For both ARI and NMI metrics, stHGCL got significantly higher scores than other competing methods, except for spCLUE. When compared with spCLUE, stHGCL still had higher median scores, but the difference was not statistically significant. Figure 2C shows that stHGCL exhibits superior performance on slice 151669, with an ARI of 0.6511 and an NMI of 0.7037. It also shows that stHGCL (ARI = 0.6511) yielded the highest ARI value, outperforming the second-ranked spCLUE (ARI = 0.6049) and third-ranked GraphST (ARI = 0.5926). Detailed clustering results for the remaining 11 slices are provided in Supplementary Figures S1–S11.

To further validate the clustering performance, we applied stHGCL to perform UMAP visualization and PAGA-based trajectory inference analysis [35] on slice 151669 (Figure 2D). This visualization analysis reveals the relationship between cluster distributions and cellular developmental trajectories. Compared to other methods, the trajectory obtained by the stHGCL method exhibits distinct layer structures. The algorithm accurately captures the developmental footprint of tissues; for instance, in human dorsolateral prefrontal cortex (DLPFC) data, it not only delineates distinct anatomical layers but also reconstructs the continuous process of cellular evolution from deep to superficial layers, which demonstrates that the data features extracted by stHGCL exhibit exceptionally high biological fidelity. Supplementary Figures S12 and S13 show the SVGs on the DLPFC slice 151669 and the top 10 GO enrichment terms. The aforementioned experimental results collectively demonstrate the significant advantages of the stHGCL method.

### 3.2. stHGCL Effectively Identifies Spatial Domains on BRCA and STARmap Datasets

**Figure 3:**
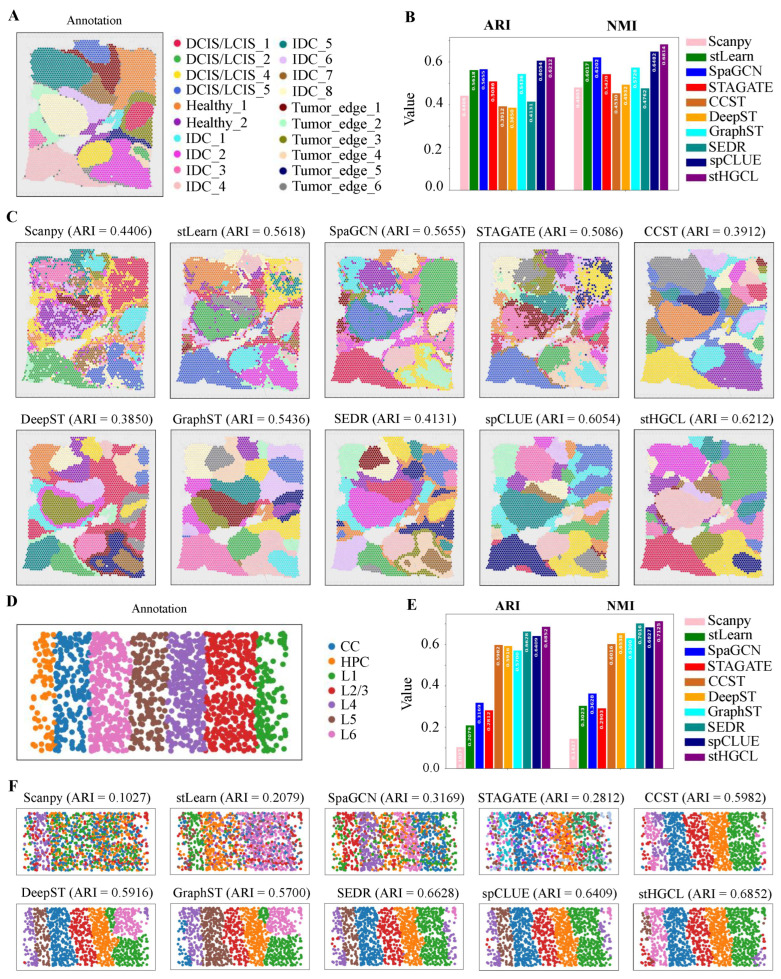
Results for spatial domain identification derived from the BRCA and STARmap datasets. (**A**) Annotation of the BRCA dataset, where DCIS denotes ductal carcinoma in situ, healthy denotes healthy tissue, IDC denotes invasive ductal carcinoma, and LCIS denotes lobular carcinoma in situ. (**B**) Bar chart presenting the values of clustering metrics ARI and NMI across various methods, with elevated bar heights indicating more favorable performance. (**C**) Clustering outputs generated by ten methods on the BRCA dataset. (**D**) Annotated spatial domain assignments corresponding to the STARmap dataset, where CC denotes corpus callosum and HPC denotes hippocampus. (**E**) Bar chart displaying the ARI and NMI clustering metric values for ten methods. (**F**) Clustering results of the ten methods applied to the STARmap dataset.

Given that spatially resolved transcriptomics technologies have emerged as indispensable tools for dissecting tumor ecosystems, we further expanded the validation of stHGCL to include a human breast cancer sample generated using the 10x Visium platform [19]. In contrast to the DLPFC dataset, this breast cancer sample exhibits more intricate spatial domain architectures and substantially higher levels of heterogeneity, which is a low-resolution dataset. Ji et al. [4] provided detailed annotations for the BRCA dataset, partitioning it into 20 spatial domains. Figure 3A illustrates the annotation of the BRCA dataset, which comprises 3798 spatial spots and 36,601 genes. Figure 3B demonstrates that stHGCL achieved the highest accuracy, with an ARI of 0.6212 and an NMI of 0.6814. It also shows that stHGCL (ARI = 0.6212) yielded the highest ARI value, outperforming the second-ranked spCLUE (ARI = 0.6054) and third-ranked SpaGCN (ARI = 0.5655). Figure 3C visualizes the clustering results of all methods.

Subsequently, to verify the efficacy of this method at single-cell resolution, we applied stHGCL to perform an in-depth analysis of the mouse visual cortex dataset derived from STARmap [6]. This dataset consists of 1020 genes across 1207 spatial spots, encompassing six distinct neocortical layers. The annotation for slice 2 is depicted in Figure 3D. As illustrated in Figure 3E,F, stHGCL attained the highest accuracy, with an ARI score of 0.6852 and an NMI score of 0.7125, both exceeding those of alternative methods.

The stHGCL method achieved the highest clustering performance on the BRCA and STARmap datasets. The spatial domains it identified are smoother, possess clear boundaries between different classes, and are more consistent with the annotations. This result indicates that stHGCL not only performs well on low-resolution datasets but also exhibits superior clustering performance on single-cell-resolution ST datasets.

### 3.3. stHGCL Effectively Identifies Spatial Domains on MOSTA and BARISTA Datasets

**Figure 4:**
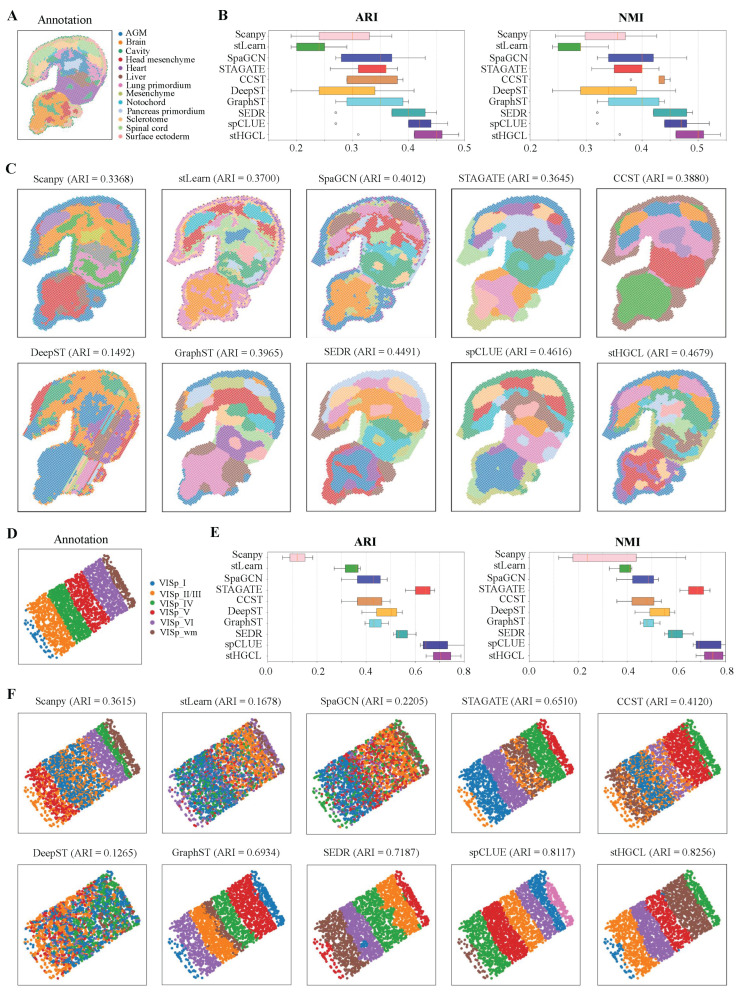
Performance of spatial domain detection on the MOSTA and BARISTA datasets. (**A**) Annotation of slice E2S2 from the E9.5 mouse embryo. (**B**) ARI and NMI scores for the five MOSTA slices. The bottom, middle, and top hinges represent the first quartile, median, and third quartile, respectively. The whiskers extend to 1.5 times the interquartile range from the hinge.Data beyond the whiskers are defined as outliers and plotted individually as circles. (**C**) Predicted spatial domains of slice E2S2 for the MOSTA dataset. (**D**) Annotation of slice 2 from the BARISTA dataset. (**E**) The ARI and NMI scores on the three BARISTA slices. (**F**) Predicted spatial domains of slice 2 from the BARISTA dataset.

Next, the clustering performance of stHGCL was tested on the MOSTA dataset, which was generated using Stereo-seq technology [8]. This dataset contains five slices from the E9.5 stage of mouse embryo, namely E1S1, E2S1, E2S2, E2S3 and E2S4, and each slice is partitioned into 12 to 14 spatial domains. For this dataset, the count of spatial spots per slice varies between 4356 and 5913, while the number of genes ranges from 23,398 to 25,568. Taking slice E2S2 as a representative case, its annotation is visualized in Figure 4A. Figure 4B demonstrates that stHGCL achieved the highest mean ARI and NMI across all five MOSTA slices, outperforming the nine alternative methods. According to Figure 4C, stHGCL demonstrated superior efficacy in delineating the head mesenchyme, pancreas primordium and spinal cord areas, achieving the highest accuracy on slice E2S2 and an ARI score of 0.4679. The clustering results of the remaining four slices are detailed in Supplementary Figures S14–S17.

Furthermore, the clustering performance of stHGCL was tested on the BARISTA dataset, which was generated using BaristaSeq technology [36]. The BARISTA dataset contains three slices isolated from the mouse cortex, namely slice 1, slice 2, and slice 3. Each slice encompasses six layers and includes transcriptomic profiles of 79 genes, with the number of capture spots spanning from 1525 to 2042. Figure 4D shows the spatial annotation for slice 2. Figure 4E displays the ARI and NMI scores of the stHGCL method and the nine other alternative methods on slice 2 of the BARISTA dataset.

As shown in Figure 4F, on slice 2, stHGCL’s annotation results and the label annotations show good consistency. stHGCL demonstrated the highest precision in spatial domain identification, yielding a leading ARI of 0.8256. The stHGCL demonstrated superior efficacy in delineating the VISp_wm and VISp_VI areas, showing a high consistency between the predicted spatial domains and the annotation. Thus, on the MOSTA dataset, the stHGCL method outperformed the other nine alternative methods. The clustering results for slice 1 and slice 3 are detailed in Supplementary Figures S18 and S19.

### 3.4. stHGCL Effectively Identifies Spatial Domains on MOB1 and MOB2 Datasets

In this subsection, the performance of the stHGCL method was evaluated on two mouse olfactory bulb (MOB) datasets, denoted as MOB1 and MOB2 [8,37]. The MOB1 and MOB2 data were generated using SlideseqV2 and Stereo-seq technologies, respectively. Given that these two datasets lack ground truth labels, we utilized the Silhouette Coefficient (SC) [38] and Calinski–Harabasz Index (CH) [39] as quantitative evaluation metrics to assess the effectiveness of the clustering results.

**Figure 5:**
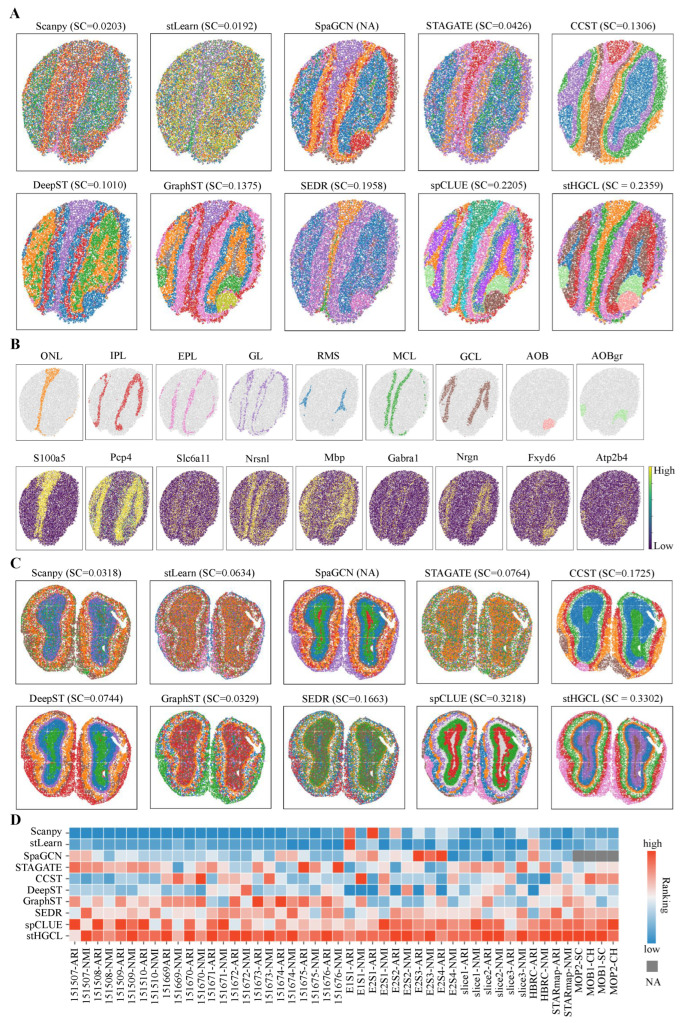
Visualization of spatial domain identification on the MOB1 and MOB2 datasets. (**A**) Spatial clustering results for the MOB1 dataset. (**B**) Expression of specific marker genes across stHGCL-identified spatial domains in MOB1. In MOB1 dataset, the domains delineated were categorized in accordance with the *Allen Brain Reference Atlas* [37] (abbreviations: AOB, AOBgr, RMS, GCL, IPL, MCL, EPL, GL and ONL). (**C**) Spatial clustering results for the MOB2 dataset. (**D**) Ranking-based performance comparison over seven single-slice datasets. Rankings range from 1 (optimal) downwards. Metrics SC and CH are assigned NA values for methods that are unable to generate low-dimensional latent representations.

Furthermore, this section adopts the *Allen Brain Reference Atlas* as the annotation benchmark for the MOB1 dataset [37]. The MOB1 annotation comprises the following anatomical components, where AOBgr denotes accessory olfactory bulb granular layer, AOB denotes accessory olfactory bulb, EPL denotes external plexiform layer, GL denotes glomerular layer, GCL denotes granule cell layer, MCL denotes mitral cell layer, IPL denotes internal plexiform layer, ONL denotes olfactory nerve layer, and RMS denotes rostral migratory stream. A comparison of the spatial domain identification accuracy and SC scores across all the evaluated methods is presented in Figure 5A. Because SpaGCN cannot obtain feature representations for spatial spots, its SC score is marked as “NA”. The stHGCL performed best in identifying the EPL, AOB and AOBgr layers. According to the SC scores, the stHGCL method obtained the highest SC scores. The CH scores of the MOB1 dataset are shown in Supplementary Figure S20. Additionally, as shown in Figure 5B, we detected differentially expressed genes (DEGs) between these spatial domains, with many of them coinciding with well-characterized marker genes. The *Allen Brain Reference Atlas* is also used as the reference labels for the MOB2 dataset [8]. Figure 5C demonstrates that stHGCL achieves the highest SC score, showing superior spatial domain recognition capability compared to alternative methods. When the SC score of SpaGCN is designated as “NA”, it is noteworthy that the spatial domains identified via stHGCL are in excellent agreement with the reference annotations. The CH scores are shown in Supplementary Figure S21.

A global view of the clustering performance for the ten methods is presented in Figure 5D, encompassing a total of seven datasets. The four clustering metrics on the single-slice datasets were converted into ranked scores, with “NA” used to mark metrics that could not be computed. When ranking the clustering metrics for each method, “1” indicates the highest rank score. Based on the ranked scores of the ten methods on the seven datasets, it can be seen that the stHGCL method ranked first or close to first on all four clustering metrics. This demonstrates that stHGCL has strong spatial clustering performance.

### 3.5. stHGCL Leverages Cross-Slice Integration for Accurate Spatial Domain Identification

**Figure 6:**
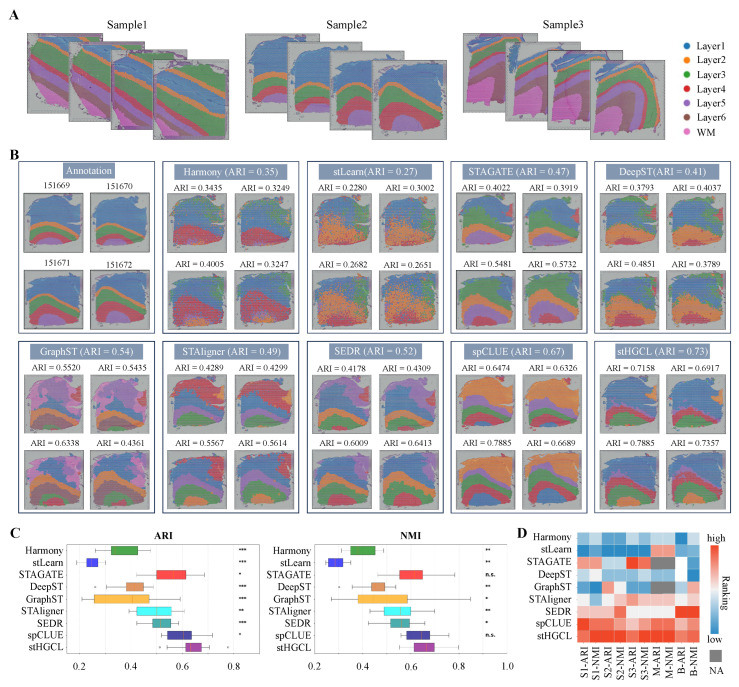
Analysis of spatial domains identified on multi-slice datasets. (**A**) Schematic of the three sample datasets from the DLPFC datasets. (**B**) Comparison of integration results for the Sample2 dataset. (**C**) Comparison of ARI and NMI scores on all 12 slices for the DLPFC dataset. Notations for statistical significance (stHGCL vs. other methods): n.s. *p* > 0.05; * *p* < 0.05; ** *p* < 0.005; *** *p* < 0.0005. The bottom, middle, and top hinges represent the first quartile, median, and third quartile, respectively. The whiskers extend to 1.5 times the interquartile range from the hinge.Data beyond the whiskers are defined as outliers and plotted individually as circles. (**D**) Comparison of ranked scores for all metrics on the three multi-slice datasets, where S1 denotes Sample1 dataset, S2 denotes Sample2 dataset, S3 denotes Sample3 dataset, M denotes MOSTA dataset, and B denotes BARISTA dataset. For STAGATE and GraphST methods that cannot perform integration with unaligned datasets (MOSTA dataset), SC and CH rankings are displayed as NA.

When processing multiple sections from the same organ, integrating the respective datasets is critical to subsequent analytical workflows. Integrating multi-slice data helps to identify consistent spatial domains across different slices. This is crucial for understanding the gene expression patterns within the slices and discovering new biological insights. To assess the performance of stHGCL on multi-slice datasets, the method was implemented on three benchmark datasets, namely DLPFC, MOSTA, and BARISTA. Specifically, the DLPFC dataset is composed of 12 slices obtained from three volunteers, with each volunteer contributing four slices; these slices are designated as Sample1 (151507–151510), Sample2 (151669–151672), and Sample3 (151673–151676), and their corresponding label annotations are presented in Figure 6A. The MOSTA dataset consists of five slices, and the BARISTA dataset consists of three slices.

We performed a systematic comparative assessment of stHGCL in conjunction with eight alternative methods on the DLPFC dataset for the purpose of verifying the performance of stHGCL in spatial domain identification. The alternative methods were Harmony, stLearn, STAGATE, DeepST, GraphST, STAligner, SEDR, and spCLUE. Figure 6B shows the clustering results of stHGCL and the eight alternative methods on Sample2. stHGCL achieved the highest ARI scores on Sample2, as well as the highest NMI scores. We visualized the integration outcomes of Sample2, which comprises four slices. The clustering results of stHGCL showed good alignment with the annotation, yielding the highest ARI and NMI scores, followed by spCLUE and GraphST. Beyond the detailed visualizations for Sample2, Supplementary Figures S22 and S23 show the clustering results of stHGCL and eight alternative methods on Sample1 and Sample3, respectively. stHGCL achieved the highest NMI values for both Sample1 and Sample3. The overall performance rankings across all the metrics confirm that stHGCL consistently attained the top or near-top positions across the three samples, enabling the effective identification of consistent spatial domains across diverse developmental phases.

Figure 6C intuitively presents the ARI and NMI scores of all the evaluated methods on the multi-slice dataset (encompassing 12 slices). Notably, stHGCL outperformed the eight alternative methods with the highest mean scores across all 12 slices, followed by spCLUE and STAGATE. For the multi-slice DLPFC dataset, we carried out the Benjamini–Hochberg test. In terms of the ARI and NMI metrics, stHGCL got significantly higher scores than other competing methods on this multi-slice DLPFC dataset. These experimental results show that stHGCL performed better in clustering than other methods across the multi-slice DLPFC datasets.

To conduct a full-scale comparison, the performance of nine alternative methods was subsequently evaluated on the MOSTA and BARISTA multi-slice datasets. Supplementary Figures S24 and S25 show the clustering results for MOSTA and BARISTA, respectively. On the MOSTA and BARISTA datasets, stHGCL also secured top or near-top rankings in terms of both ARI and NMI scores.

The ranked scores of the ARI and NMI metrics for the nine methods are presented in Figure 6D. The stHGCL method ranked first or close to first on each dataset. This reflects that stHGCL can effectively cluster multi-slice data from different developmental stages.

We evaluated the computational efficiency of stHGCL in terms of runtime and memory usage, comparing it with nine single-slice alternative methods (Supplementary Figure S26) and eight multi-slice alternative methods (Supplementary Figure S27). The results demonstrate that stHGCL achieves well-balanced performance, positioning itself in the median range across all the tested benchmarks for both single-slice and multi-slice data integration.

### 3.6. Ablation Study

**Figure 7:**
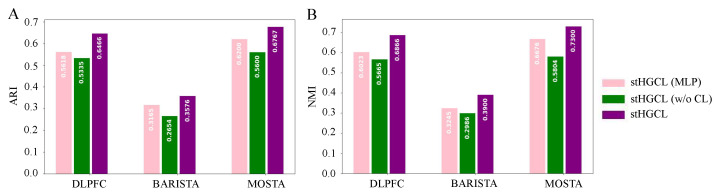
Bar charts illustrating the performance of stHGCL and its variants on the three multi-slice datasets, with the bars color-coded according to the corresponding stHGCL variant. For multi-slice datasets, displayed metrics represent the average values per multi-slice dataset. (**A**) ARI score. (**B**) NMI score.

To evaluate the individual contributions of the HetGNN and the contrastive learning components, we analyzed several stHGCL variants. We implemented stHGCL (multi-layer perceptron, MLP) by replacing the heterogeneous graph neural network (HetGNN) with a multi-layer perceptron of equivalent depth and dimensionality. Another variant, stHGCL (w/o CL), was developed by omitting the contrastive learning objective. The performance comparisons across ARI and NMI metrics on multi-slice data, visualized in Figure 7, confirm that the integrated stHGCL framework consistently yields the best results.

The ablation study confirms that the stHGCL’s overall efficacy is driven by the synergistic effects of the HetGNN and its contrastive learning component.

### 3.7. Parameter Analysis

**Figure 8:**
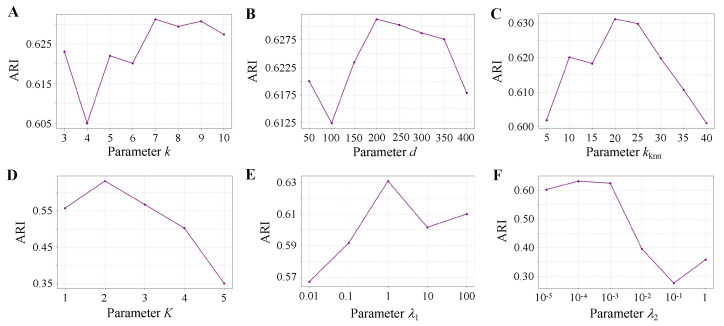
Sensitivity analysis of model parameters on the BRCA dataset. (**A**) Spatial neighbors *k*. (**B**) PCA dimension *d*. (**C**) K-nearest neighbors $k_{\mathrm{knn}}$. (**D**) Network layer *K*. (**E**) Hyperparameter $\lambda_{1}$. (**F**) Hyperparameter $\lambda_{2}$.

To analyze the influence of key parameters in stHGCL, we evaluate the initial spatial neighbors *k*, PCA dimension *d*, k-nearest neighbors $k_{\mathrm{knn}}$, network layer *K*, and hyperparameters $\lambda_{1}$ and $\lambda_{2}$. Figure 8 shows the ARI scores achieved by stHGCL across a broad range of parameter settings.

For spatial neighbors k∈{3,4,5,6,7,8,9,10}, the highest ARI occurs at k=7 (Figure 8A). For PCA dimension d∈{50,100,150,200,250,300,350,400}, the optimal value is d=200 (Figure 8B). For the k-nearest neighbors $k_{\mathrm{knn}}\in \left\{5,\;10,\;15,\;20,\;25,\;30,\;35,\;40\right\}$, the best performance occurs at $k_{\mathrm{knn}}=20$ (Figure 8C). For the network layer K∈{1,2,3,4,5}, the peak ARI is achieved at K=2 (Figure 8D). The hyperparameters $\lambda_{1}\in \left\{0.01,\;0.1,\;1,\;1,\;100\right\}$ and $\lambda_{2}\in \left\{10^{-5},\;10^{-4},\;10^{-3},\;10^{-2},\;10^{-1},\;1\right\}$ are tested, and $\lambda_{1}=1$ (Figure 8E) and $\lambda_{2}=10^{-3}$ (Figure 8F) demonstrate the best performance.

## 4. Discussion

Identifying spatial domains represents a fundamental challenge in interpreting SRT data. Herein, we introduce stHGCL, a novel computational method that employs a heterogeneous spot–gene graph architecture to achieve robust domain identification across both single-slice and multi-slice datasets. The framework encompasses three core functional modules: (1) stHGCL employs a heterogeneous graph neural network on the constructed spot–gene heterogeneous graph to extract high-order structural information, directly learning spot–gene relationships and indirectly capturing spot–spot relationships; (2) stHGCL uses a dual-stage encoder consisting of a multi-layer HetGNN leveraging LightGCN and a subsequent GCN to learn preliminary spot embeddings and final gene embeddings, as well as to refine the final spot embeddings by aggregating local spatial locations from a spot-neighborhood graph; (3) stHGCL introduces a contrastive learning loss module, which constrains spot embeddings via spot-neighborhood relationships generated by the encoder, thus improving integration performance. Comprehensive ablation experiments verify the effectiveness of stHGCL and demonstrate its robustness.

We evaluated the performance of stHGCL across a panel of analytical tasks spanning both single-slice and multi-slice experimental paradigms. For the single-slice datasets, stHGCL was subjected to systematic benchmarking against nine cutting-edge computational methods using datasets derived from a diverse set of technological platforms, including 10x Visium, BaristaSeq, STARmap, Slide-seqV2, and Stereo-seq. Relative to these alternative methods, stHGCL attained the highest or near-highest overall performance and rankings across all the tested experimental platforms, thereby demonstrating its exceptional effectiveness in spatial domain delineation. For multi-slice analysis, stHGCL exhibited leading performance among eight alternative methods on three complex datasets (DLPFC, MOSTA and BARISTA), which validates its capacity to integrate spatially resolved transcriptomics data across multiple slices. On each multi-slice dataset, stHGCL outperformed alternative methods by securing the top or near-top rankings in ARI and NMI scores across all the slices. Furthermore, a global evaluation of multi-slice benchmarks confirms that stHGCL frequently occupies the highest performance rankings. These findings underscore the model’s proficiency in maintaining spatial consistency and alleviating slice-related discrepancies across various developmental phases.

Despite the robust performance across diverse platforms, we acknowledge certain limitations of stHGCL that warrant further consideration. First, in tissues characterized by highly diffuse architectures or high cellular intermixing without distinct morphological boundaries, our neighborhood-driven contrastive learning module may lead to over-smoothing of fine-grained biological transitions. Second, for low-resolution datasets where each capture unit encompasses a high diversity of cell types, the current heterogeneous graph framework may struggle to resolve the underlying biological complexity without integrated single-cell deconvolution. Furthermore, the model’s efficacy is inherently linked to data quality; extreme gene sparsity or low spot density can result in a sparse heterogeneous graph, potentially limiting the capture of high-order structural information. Lastly, as the scale of SRT datasets grows to tens of thousands of spots, the memory footprint for constructing large-scale adjacency matrices and the $O(B^{2})$ complexity of identifying inter-slice MNNs may present computational bottlenecks.

For SRT data, a promising extension lies in deeper integration of histological image information. As many SRT technologies now provide histological images alongside spatial coordinates, and prior methods (e.g., stLearn [15], SpaGCN [13], and DeepST [20]) have validated that image features substantially boost clustering and domain identification, stHGCL can incorporate histological features as an optional input view. Fusing these with gene expression-derived embeddings could enhance spatial domain detection. Another key direction is integrating SRT with multi-modal data (e.g., spatial ATAC-seq and spatial proteomics). stHGCL’s contrastive learning framework inherently supports such expansion: constructing new omics-specific views and enforcing consistent spot embeddings across modalities would enable robust multi-omics fusion, further improving the unification of single-slice and multi-slice data analysis. Lastly, integration with high-resolution scRNA-seq data merits exploration. Given that many SRT spots still cover multiple spots, adapting stHGCL to jointly process scRNA-seq and SRT data could guide cell-type deconvolution of spatial spots and map single-cell data back to spatial domains. This would strengthen the complementarity between the two data types, advancing both single-slice domain refinement and multi-slice cross-sample integration.

## 5. Conclusions

In summary, we propose stHGCL, a unified spatial domain detection method for both single-slice and multi-slice transcriptomic datasets. By utilizing a spot–gene heterogeneous graph, the model uses a dual encoder to learn spot embeddings that effectively capture high-order structural information. Furthermore, the integration of a contrastive learning objective leverages encoder-derived neighborhood structures to refine spot embeddings and boost cross-sample alignment. Extensive benchmarking across a diverse set of technological platforms and experimental settings confirms that stHGCL delivers superior domain detection accuracy. Ultimately, stHGCL offers a robust and scalable solution for the complex integration and analysis of spatial transcriptomic data.

## Data Availability

The human DLPFC dataset can be accessed in the spatialLIBD package http://spatial.libd.org/spatialLIBD/ [32]. The BRCA dataset and the MOB2 dataset can be downloaded from https://github.com/JinmiaoChenLab/SEDR_analyses/tree/master/data [19]. The STARmap dataset can be downloaded from https://www.dropbox.com/sh/f7ebheru1lbz91s/AADm6D54GSEFXB1feRy6OSASa/visual_1020/20180505_BY3_1kgenes?dl=0&subfolder_nav_tracking=1 [6]. The MOSTA dataset is available at https://db.cngb.org/stomics/mosta/ [8]. The BARISTA dataset can be downloaded from http://sdmbench.drai.cn [36]. The MOB1 dataset generated by Slide-seqV2 platforms can be accessed from https://singlecell.broadinstitute.org/single_cell/study/SCP815 [37]. The code scripts utilized in this study can be accessed at https://github.com/Xia-xia-li/stHGCL all accessed on 1 February 2026.

## Supplementary Materials

The following supporting information can be downloaded at: https://www.mdpi.com/article/10.3390/genes17030310/s1, Figures S1–S27, Tables S1 and S2, and Supplementary Methods.
