# Supplementary material for: Leveraging Spot–Gene Heterogeneous Graphs for Unified Spatially Resolved Transcriptomics Domain Detection on Single-Slice and Multi-Slice Data
Source: Genes (Basel). 2026 Mar 7;17(3):310. doi: 10.3390/genes17030310 (PMC13025579; doi:10.3390/genes17030310)
Supplement: Supplementary file 1 [file genes-17-00310-s001.zip › genes-4129503-supplementary.pdf]

Supplementary Information

"Leveraging spot-gene heterogeneous graphs for unified spatially resolved transcriptomics domain detection on single-slice and multi-slice data"

Table of contents

1. Supplementary Methods 3

1.1 Hyperparameter settings of stHGCL 3

1.2 Evaluation metrics 4

1.3 Combined score 6

1.4 Baseline and competing methods 7

1.5 Datasets 9

2. References 11

3. Supplementary Figures 14

4. Supplementary Tables 25

1. Supplementary Methods

1.1 Hyperparameter settings of stHGCL

The stHGCL software is available at *https://github.com/Xia-xia-li/stHGCL*, and was applied as described in its tutorial.

Data Preprocessing. For each spatial transcriptomics (SRT) data, the raw gene expression matrix is log-transformed and normalized using the Scanpy package [1]. We select the top 2,000 highly variable genes (HVGs) to maintain biological signal while reducing noise. Then, principal component analysis (PCA) is performed to reduce the gene expression dimensionality to 200 components, which serve as the primary model input.

Heterogeneous Graph. We construct a spot-gene heterogeneous graph where an edge represents the expression of gene j in spot i. During training, we randomly select a sample rate of 10% of existing edges as positive samples and an equal number of non-existing edges as negative samples to construct subgraphs for the reconstruction task.

Spatial Graph. A spatial neighborhood graph is built using the Euclidean distance between spots based on their spatial coordinates. For each spot, we identify the $k = 7$ nearest neighbors to capture local spatial context, a parameter value that demonstrated consistent performance across various SRT technologies.

Dual-Stage Encoder. The encoder consists of dual-stages: a LightGCN stage with $k = 2$ layers to capture high-order spot-gene structural information, followed by a GCN stage with 2 layers for local spatial information aggregation. The final feature embedding dimension is set to 64.

Contrastive Learning Module. For multi-slice integration, we utilize mutual nearest neighbors identified in the canonical correlation analysis space as anchor-positive pairs to mitigate batch effects. Within a single batch, positive samples are identified via k-nearest neighbors in PCA space, while negative samples are

randomly selected from different pre-clusters obtained through Louvain method [2]. The triplet margin is set to $l = 0.5$ to ensure discriminative embedding learning.

Optimization. The stHGCL framework employs a staged learning rate strategy using the Adam optimizer. For multi-slice integration, the learning rate is set to 0.001 to ensure stable convergence across batches. In single-slice tasks, the learning rate is set to 0.01.

Clustering. For single-slice analysis, only the single-slice heterogeneous graph and neighborhood graph are needed. Due to the sparse number of spots in single slices, we use K-means method [3] for stable clustering. For multi-slice patial transcriptomics data, the final embeddings are clustered using the mclust method [4] to achieve spatial domain detection. For applications presented in this work, the number of clusters was set to the annotated layer or cell type number.

1.2 Evaluation metrics

To comprehensively and objectively evaluate the clustering performance of stHGCL, for datasets with annotated spatial regions, we measure model accuracy by quantifying the consistency between predicted labels and ground truth labels. The metrics employed include the Adjusted Rand Index (ARI) [5], Normalized Mutual Information (NMI) [6]. Conversely, for datasets without ground truth annotations, we reflect the intrinsic quality of the clustering structure by evaluating intra-cluster compactness and inter-cluster separation, specifically utilizing the Silhouette Coefficient (SC) [7] and the Calinski-Harabasz Index (CH) [8].

ARI. ARI serves as a classic metric for measuring the similarity between two data partitions. By introducing a correction for random chance, it avoids the issue of inflated scores as the number of clusters increases. Let $U = \{u_1, \ldots, u_R\}$ denote the ground truth class partition and $V = \{v_1, \ldots, v_C\}$ denote the model-predicted clustering partition. The specific calculation formula for ARI is:

$$ARI = \frac{\sum_{i,j}\binom{n_{ij}}{2} - \left[\sum_i\binom{a_i}{2}\sum_j\binom{b_j}{2}\right] / \binom{n}{2}}{\frac{1}{2}\left[\sum_i\binom{a_i}{2} + \sum_j\binom{b_j}{2}\right] - \left[\sum_i\binom{a_i}{2}\sum_j\binom{b_j}{2}\right] / \binom{n}{2}}$$

where n represents the total number of samples, n_{ij} denotes the number of samples belonging to both the true class u_i and the predicted cluster v_j (i.e., elements in the contingency matrix), $a_i = \sum_j n_{ij}$ is the total number of samples in the true class u_i, and $b_j = \sum_i n_{ij}$ is the total number of samples in the predicted cluster v_j. The notation $\binom{\cdot}{2}$ represents the binomial coefficient calculation. This metric ranges from $[-1,1]$, where a higher value indicates more accurate clustering results.

NMI. NMI evaluates clustering quality from an information-theoretic perspective. It quantifies the shared information between ground truth labels and predicted clusters based on Shannon entropy, utilizing a geometric mean for normalization. Its detailed calculation formula is defined as:

$$NMI(U,V) = \frac{-2\sum_{i=1}^{R}\sum_{j=1}^{C} n_{ij}\log\left(\frac{n \cdot n_{ij}}{a_i b_j}\right)}{\sum_{i=1}^{R} a_i \log\left(\frac{a_i}{n}\right) + \sum_{j=1}^{C} b_j \log\left(\frac{b_j}{n}\right)}$$

where R and C represent the number of categories in the ground truth and predicted clusters, respectively. The definitions of n_{ij}, a_i, and b_j are consistent with those in ARI, representing the number of overlapping samples, total samples in the true class, and total samples in the predicted cluster, respectively; n is the total sample size. The numerator measures the difference between the joint distribution of U and V and the product of their marginal distributions via logarithmic operations, while the denominator represents the sum of their entropies, ensuring the result falls within the $[0,1]$ interval.

SC. For unannotated data, the SC evaluates clustering validity by combining intra-cluster cohesion and inter-cluster separation. For any sample point i in the dataset, its Silhouette Coefficient is defined as:

$$SC = \frac{1}{n}\sum_{i=1}^{n}\frac{b(i) - a(i)}{\max\{a(i), b(i)\}}$$

where $a(i)=\frac{1}{|C_I|-1}\sum_{j\in C_I, j\neq i} d(i,j)$ represents the mean distance between sample i and all other samples in the same cluster C_I (cohesion), and $b(i)=\min_{J\neq I}\left(\frac{1}{|C_J|}\sum_{j\in C_J} d(i,j)\right)$ represents the mean distance between sample i and all samples in its nearest neighboring cluster C_J (separation), with $d(i,j)$ denoting the Euclidean distance. An SC mean closer to 1 implies that the sample matches its own cluster well and is well-separated from other clusters.

CH. The CH assesses the clustering model by comparing the trace of the inter-cluster dispersion matrix to the trace of the intra-cluster dispersion matrix. For n samples grouped into k clusters, the specific calculation formula for the CH index is:

$$CH=\frac{\mathrm{Tr}(B_k)}{\mathrm{Tr}(W_k)}\cdot\frac{n-k}{k-1}=\frac{\sum_{q=1}^{k} n_q \| c_q - c\|^2}{\sum_{q=1}^{k}\sum_{x\in C_q}\| x-c_q\|^2}\cdot\frac{n-k}{k-1}$$

where B_k is the inter-cluster dispersion matrix, W_k is the intra-cluster dispersion matrix, and $\mathrm{Tr}(\cdot)$ denotes the trace of a matrix. In the expanded form, n_q is the number of samples in the q-th cluster C_q, c_q is the center point of cluster C_q, c is the global center point of all data, and x represents a single sample data point. A higher CH score indicates larger distances between clusters (numerator) and smaller distances within clusters (denominator), reflecting superior clustering performance.

1.3 Combined score

In the context of Gene Ontology (GO) enrichment analysis, the Combined Score is a calculated metric used to rank the significance of enriched biological terms. It is typically determined by the following formula:

$$c = \ln(q) \cdot z,$$

where q is the q-value computed using Fisher's exact test, which indicates the probability that the observed enrichment occurred by chance; z is the z-score, which represents the deviation from the expected rank.

A higher Combined Score signifies a more statistically robust and biologically relevant enrichment of that specific GO term within the identified spatially variable genes (SVGs).

1.4 Baseline and competing methods

To evaluate the clustering performance of stHGCL, we benchmarked it against 11 state-of-the-art methods on various datasets. Scanpy served as the baseline method as it solely relies on gene expression data. The other methods incorporated spatial information, including Scanpy [1], Harmony [9], stLearn [10], SpaGCN [11], STAGATE [12], CCST [13], DeepST [14], GraphST [15], STAligner [16], SEDR [17], and spCLUE [18]. Below is a brief description of the usage of each method:

Scanpy [1]. Scanpy served as the baseline method as it solely relies on gene expression data without incorporating spatial coordinates. It employs Principal Component Analysis (PCA) for dimensionality reduction and utilizes the Louvain algorithm for clustering spots based on their expression profiles. We ran Scanpy using the standard pipeline with default parameters.

Harmony [9]. Harmony employs a maximum diversity clustering strategy combined with iterative correction to integrate datasets. It projects cells into a shared embedding space to correct for batch effects across different slices. Following the documentation, we used the latent representations generated by Harmony for downstream clustering and visualization.

stLearn [10]. stLearn leverages histology images and spot positions to construct the Spatial Morphological gene Expression (SME) weighting matrix for SME-based gene normalization. Following the tutorial, we ran stLearn with default parameters on both spot-based and single-cell spatial transcriptomics data.

SpaGCN [11]. SpaGCN integrates spatial locations, histological similarities, and gene expression to build a graph, using a graph convolutional network (GCN) for clustering spots. Following tutorial recommendations, we cropped a 40x40 pixel

snapshot from the H&E image and set the adjacency matrix with histology set to True. The GCN model was trained for 200 epochs with a learning rate of 0.05.

STAGATE [12]. STAGATE combines an autoencoder with graph attention to learn latent representations. For preprocessing, we used default settings of 3000 top variable genes, normalized total expression of each spot to 10000, followed by log transformation. The STAGATE model was then run with default parameters as suggested in the vignettes.

CCST [13]. CCST utilizes a Deep Graph Infomax (DGI) approach to learn node representations. It constructs a graph to model the relationship between spots and integrates gene expression with spatial information to identify spatial domains. The model was executed using the default settings provided in the original study.

DeepST [14]. DeepST combines morphological information, spatial distance, and gene expression to generate an enhanced gene expression matrix, utilizing a variational graph autoencoder to extract latent representations. We ran DeepST to obtain latent representations with 200 PCs, keeping other parameters at default settings.

GraphST [15]. GraphST employs graph contrastive learning methods like Deep Graph Infomax (DGI) for learning latent representations of capture spots. The GraphST model was run with default parameters as suggested in the vignettes.

STAligner[16]. STAligner integrates spatial transcriptomics data by employing a graph attention autoencoder combined with a mutual nearest neighbor graph. This framework allows for the alignment of spots across different slices to identify unified spatial domains. We ran the model with default parameters as suggested in the vignettes.

SEDR [17]. SEDR uses variational autoencoders and masked self-supervised learning for representation learning and performs batch integration to some extent by constructing a large graph. We used SEDR for clustering single slices and integrating slices from the same sample, running the model with default parameters as suggested in the vignettes.

spCLUE [18]. spCLUE leverages a multi-view graph network enhanced by contrastive learning and an attention mechanism to decipher spatial domains. It integrates multiple views of the data to learn robust latent representations for spots. We followed the tutorial recommendations to run spCLUE on the datasets.

1.5 Datasets

Datasets used in this study are all publicly available and were sequenced by different platforms, including 10x Visium, BaristaSeq, SlideSeq-V2 and Stereo-seq. A brief summary of these datasets are provided in Supplementary Table S2.

DLPFC. The DLPFC dataset was sequenced by the 10x Visium technology. The data is accessible from the spatialLIBD package (*http://spatial.libd.org/spatialLIBD/*).

This dataset consists of 12 slices from 3 adult samples with 4 adjacent slices from each sample:

■ Sample1: 151507,151508,151509, 151510;

■ Sample2: 151669,151670,151671, 151672;

■ Sample3: 151673,151674,151675, 151676.

Five spatial domains are annotated in 151669, 151670, 151671 and 151672, and seven spatial domains are annotated in the other 8 slices.

BRCA. The BRCA dataset was sequenced by the 10x Visium technology. The data is accessible at *https://www.10xgenomics.com/* and was annotated into 20 spatial domains in the SEDR work. The data and the corresponding labels are available at *https://github.com/JinmiaoChenLab/SEDRanalyses/tree/master/data*.

STARmap. The STARmap dataset *https://www.dropbox.com/ sh/f7ebheru1lbz91s/AADm6D54GSEFXB1feRy6OSASa/visual_1020/20180505_BY3_1kgenes?dl=0&subfolder_nav_tracking=1.*

MOSTA. The MOSTA dataset was sequenced by the Stereo-seq technology. It consists of five mouse embryo slices at the developmental stage of E9.5. The MOSTA dataset is available at *https://db.cngb.org/stomics/mosta/.*

BARISTA. The BARISTA dataset was sequenced by the BaristaSeq technology. It consists of three slices of mouse primary visual cortex samples. The data is available at *http://sdmbench.drai.cn*.

MOB1. The MOB1 dataset was sequenced by the Slide-seqV2 technology. The data is available at the Broad Institute Single Cell Portal at *https://singlecell.broadinstitute.org/singlecell/study/SCP815/highly-sensitive-spatial-transcriptomics-at-near-cellular-resolution-with-slide-seqv2#study-summary.*

MOB2. The MOB2 dataset was sequenced by Stereo-seq technology. The data is available at *https://github.com/JinmiaoChenLab/SEDRanalyses/tree/master/data.*

2. References

1. Wolf F. A.; Angerer P.; Theis F.J. SCANPY: large-scale single-cell gene expression data analysis. *Genome Biol.* **2018**, *19*, 15.
2. Blondel V.D.; Guillaume J.L.; Lambiotte R,; Lefebvre, E. Fast unfolding of communities in large networks. *J. Stat. Mech-Theory E.* **2008**, *2008,* P10008.
3. Likas, A.; Vlassis, N.; Verbeek, J.J. The global k-means clustering algorithm. *Pattern Recogn*. **2003**, *36*, 451-461.
4. Fraley, C.; Raftery, A.E. Enhanced model-based clustering, density estimation, and discriminant analysis software: MCLUST. *J. Classif.* **2003**, *20*, 263–286.
5. Hubert, L.; Arabie, P. Comparing partitions. *J. Classif.* **1985**, *2*, 193–218.
6. Strehl, A.; Ghosh, J. Cluster ensembles – a knowledge reuse framework for combining multiple partitions. *J. Mach. learn. Res.* **2002**, *3*, 583–617.
7. Rousseeuw, P.J. Silhouettes: a graphical aid to the interpretation and validation of cluster analysis. *J. Comput. Appl. Math.* **1987**, *20*, 53–65.
8. Caliński, T.; Harabasz, J. A dendrite method for cluster analysis. *Commun. Stat.* **1974**, *3*, 1–27.
9. Korsunsky, I.; Millard, N.; Fan, J.; Slowikowski, K.; Zhang, F.; Wei, K.; Baglaenko, Y.; Brenner, M.; Loh, P.; Raychaudhuri, S. Fast, sensitive and accurate integration of single-cell data with harmony. *Nat. Methods* **2019**, *16*, 1289–1296.
10. Pham, D.; Tan, X.; Xu, J.; Grice, L.F.; Lam, P.Y.; Raghubar, A.; Vukovic, J.; Ruitenberg, M.J.; Nguyen, Q. stLearn: integrating spatial location, tissue

morphology and gene expression to find cell types, cell-cell interactions and spatial trajectories within undissociated tissues. *bioRxiv* **2020**, 2020-05.

11. Hu, J.; Li, X.; Coleman, K.; Schroeder, A.; Ma, N.; Irwin, D.J.; Lee, E.B.; Shinohara, R.T.; Li, M. SpaGCN: Integrating gene expression, spatial location and histology to identify spatial domains and spatially variable genes by graph convolutional network. *Nat. Methods* **2021**, *18*, 1342–1351.
12. Dong, K.; Zhang, S. Deciphering spatial domains from spatially resolved transcriptomics with an adaptive graph attention auto-encoder. *Nat. Commun.* **2022**, *13*, 1739.
13. Li, J.; Chen, S.; Pan, X.; Yuan, Y.; Shen, H.B. Cell clustering for spatial transcriptomics data with graph neural networks. *Nat. Comput. Sci.* **2022**, *2*, 399–408.
14. Xu, C.; Jin, X.; Wei, S.; Wang, P.; Luo, M.; Xu, Z.; Yang, W.; Cai, Y.; Xiao, L.; Lin, X.; et al. DeepST: identifying spatial domains in spatial transcriptomics by deep learning. *Nucleic Acids Res.* **2022**, *50*, e131.
15. Long, Y.; Ang, K.S.; Li, M.; Chong, K.L.K.; Sethi, R.; Zhong, C.; Xu, H.; Ong, Z.; Sachaphibulkij, K.; Chen, A.; et al. Spatially informed clustering, integration, and deconvolution of spatial transcriptomics with GraphST. *Nat. Commun.* **2023**, 14, 1155.
16. Zhou, X.; Dong, K.; Zhang, S. Integrating spatial transcriptomics data across different conditions, technologies and developmental stages. *Nat. Comput. Sci.* **2023**, *3*, 894–906.

17. Xu, H.; Fu, H.; Long, Y.; Ang, K.S.; Sethi, R.; Chong, K.; Li, M. Uddamvathanak, R.; Lee, H.K; Ling, J.; et al. Unsupervised spatially embedded deep representation of spatial transcriptomics. *Genome Med.* **2024**, *16*, 12.
18. Wang, X.; Li, W.V.; Li, H. spCLUE: a contrastive learning approach to unified spatial transcriptomics analysis across single-slice and multi-slice data. *Genome Biol.* **2025**, *26*, 177.

3. Supplementary Figures

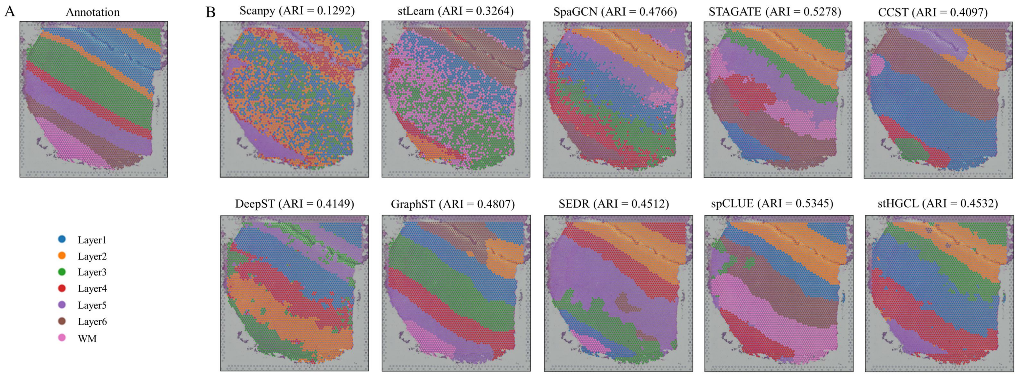

Figure S1. Visualization of different methods' clustering results on DLPFC slice 151507. (A) Annotation layer structure on slice 151507. (B) Domain identification on slice 151507 by stHGCL and nine baseline methods.

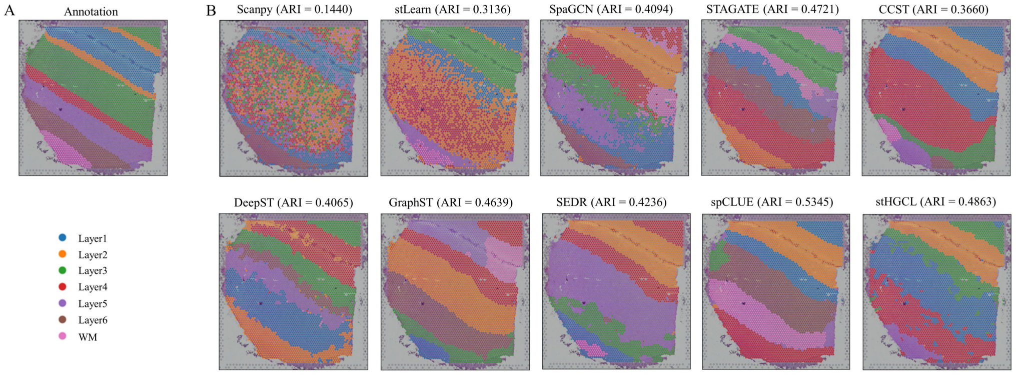

Figure S2. Visualization of different methods' clustering results on DLPFC slice 151508. (A) Annotation layer structure on slice 151508. (B) Domain identification on slice 151508 by stHGCL and nine baseline methods.

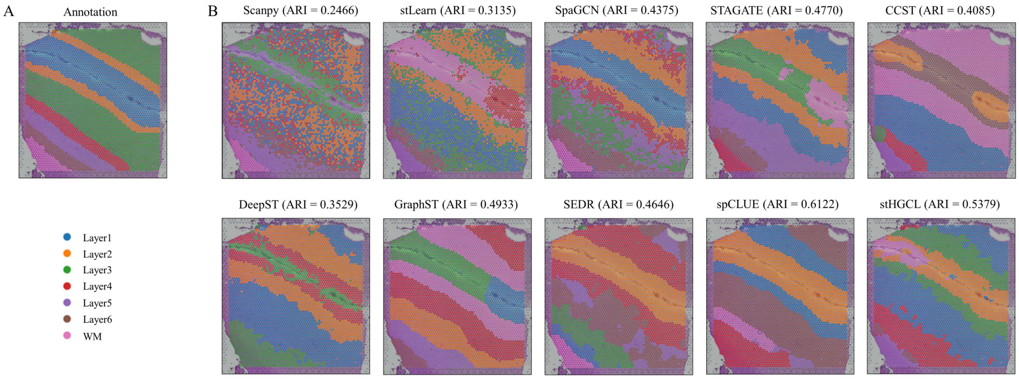

Figure S3. Visualization of different methods' clustering results on DLPFC slice 151509. (A) Annotation layer structure on slice 151509. (B) Domain identification on slice 151509 by stHGCL and nine baseline methods.

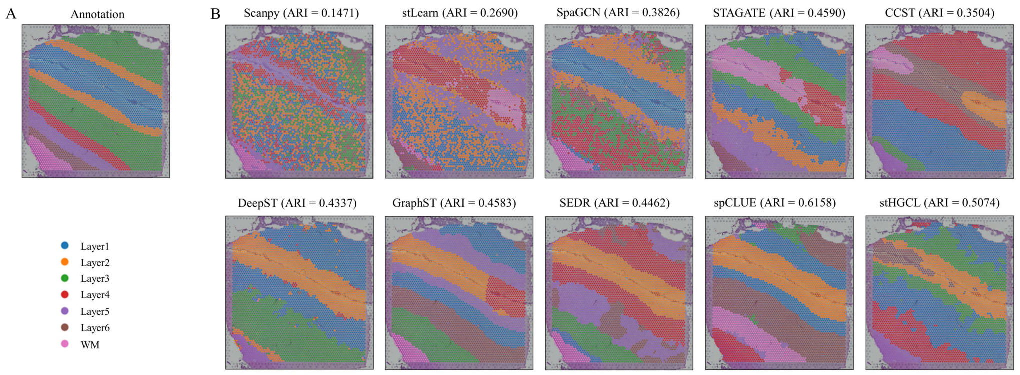

Figure S4. Visualization of different methods' clustering results on DLPFC slice 151510. (A) Annotation layer structure on slice 151510. (B) Domain identification on slice 151510 by stHGCL and nine baseline methods.

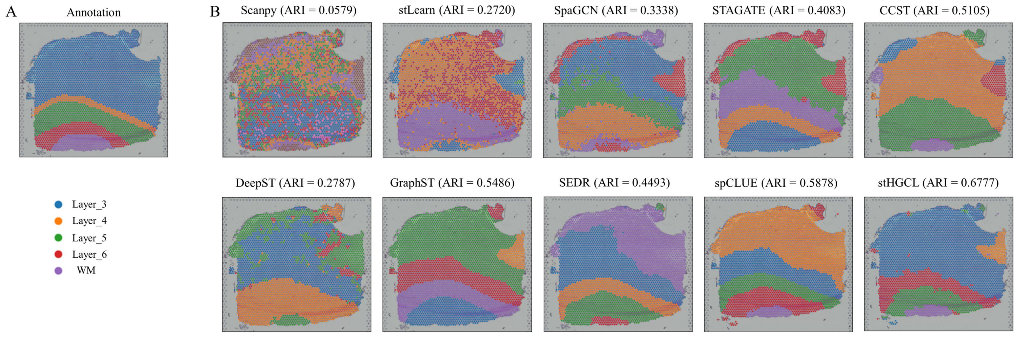

Figure S5. Visualization of different methods' clustering results on DLPFC slice 151670. (A) Annotation layer structure on slice 151670. (B) Domain identification on slice 151670 by stHGCL and nine baseline methods.

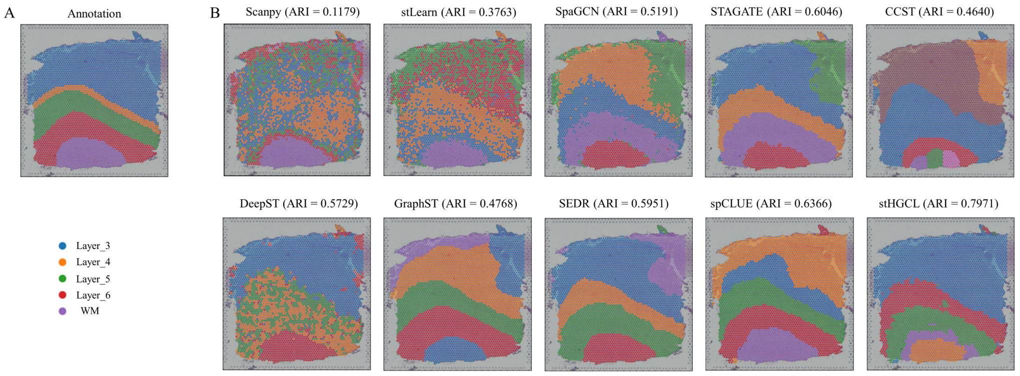

Figure S6. Visualization of different methods' clustering results on DLPFC slice 151671. (A) Annotation layer structure on slice 151671. (B) Domain identification on slice 151671 by stHGCL and nine baseline methods.

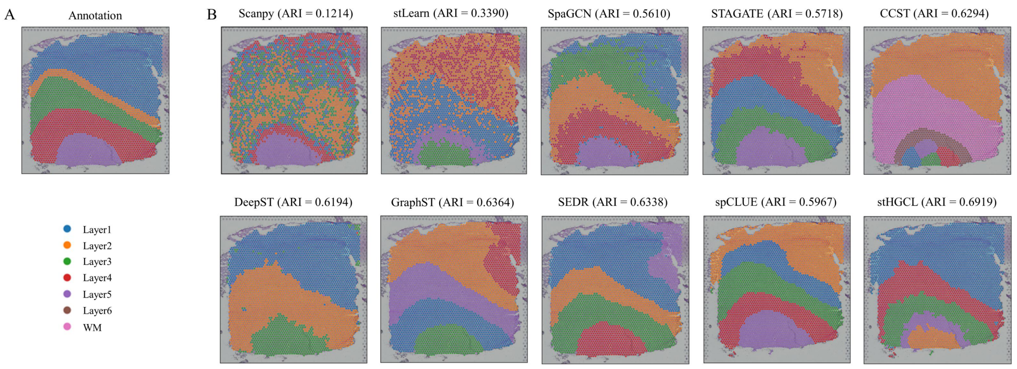

Figure S7. Visualization of different methods' clustering results on DLPFC slice 151672. (A) Annotation layer structure on slice 151672. (B) Domain identification on slice 151672 by stHGCL and nine baseline methods.

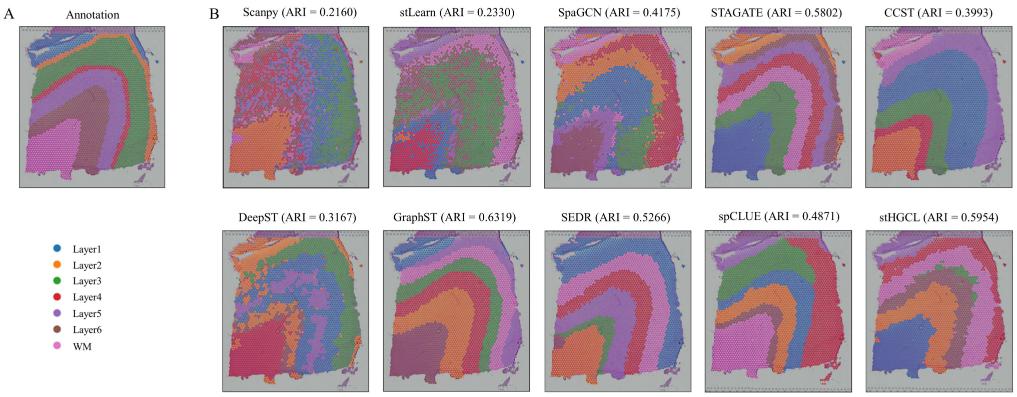

Figure S8. Visualization of different methods' clustering results on DLPFC slice 151673. (A) Annotation layer structure on slice 151673. (B) Domain identification on slice 151673 by stHGCL and nine baseline methods.

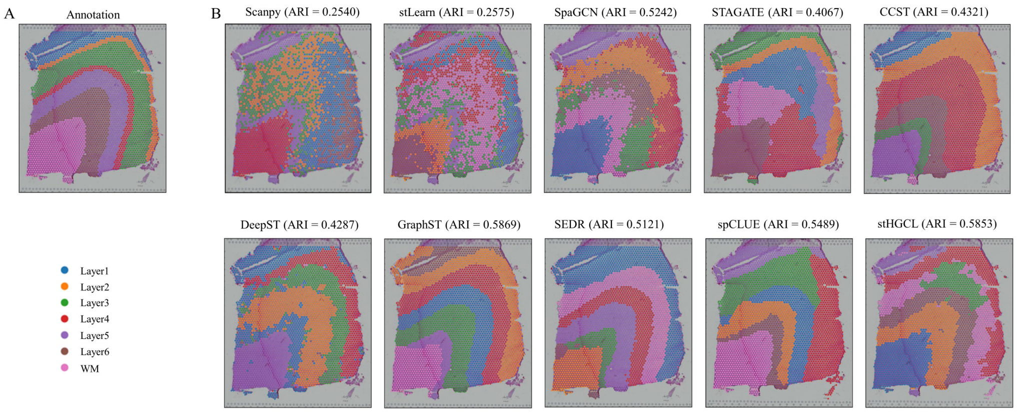

Figure S9. Visualization of different methods' clustering results on DLPFC slice 151674. (A) Annotation layer structure on slice 151674. (B) Domain identification on slice 151674 by stHGCL and nine baseline methods.

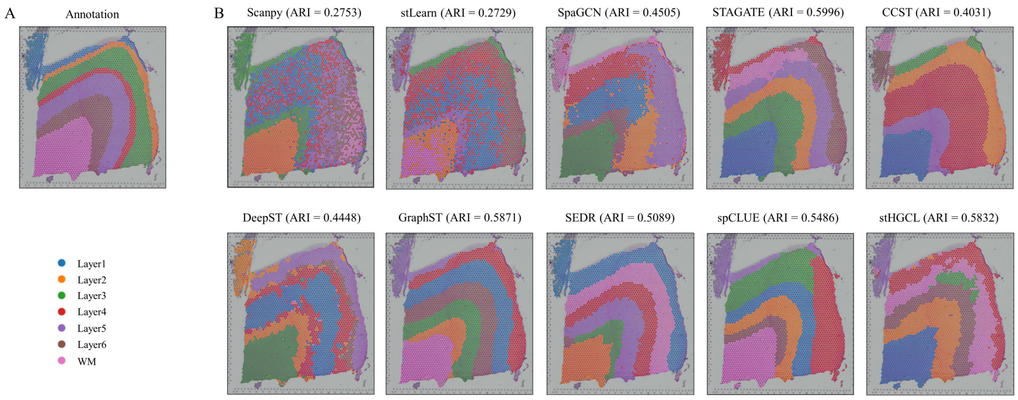

Figure S10. Visualization of different methods' clustering results on DLPFC slice 151675. (A) Annotation layer structure on slice 151675. (B) Domain identification on slice 151675 by stHGCL and nine baseline methods.

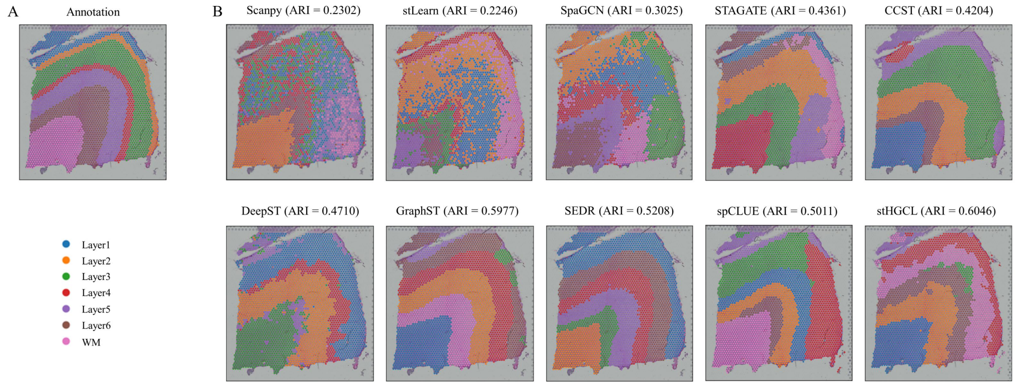

Figure S11. Visualization of different methods' clustering results on DLPFC slice 151676. (A) Annotation layer structure on slice 151676. (B) Domain identification on slice 151676 by stHGCL and nine baseline methods.

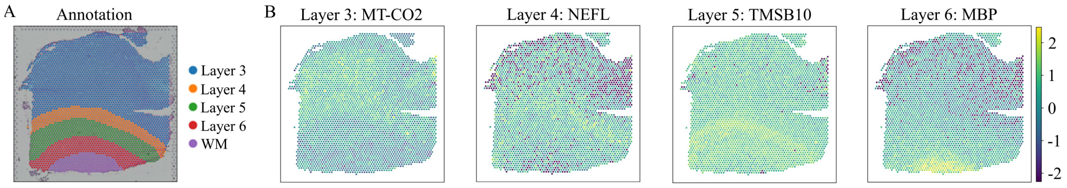

Figure S12. SVGs on the DLPFC slice 151669. (A) Annotation layer structure on slice 151669. (B) Spatial expression patterns of SVGs detected by stHGCL.

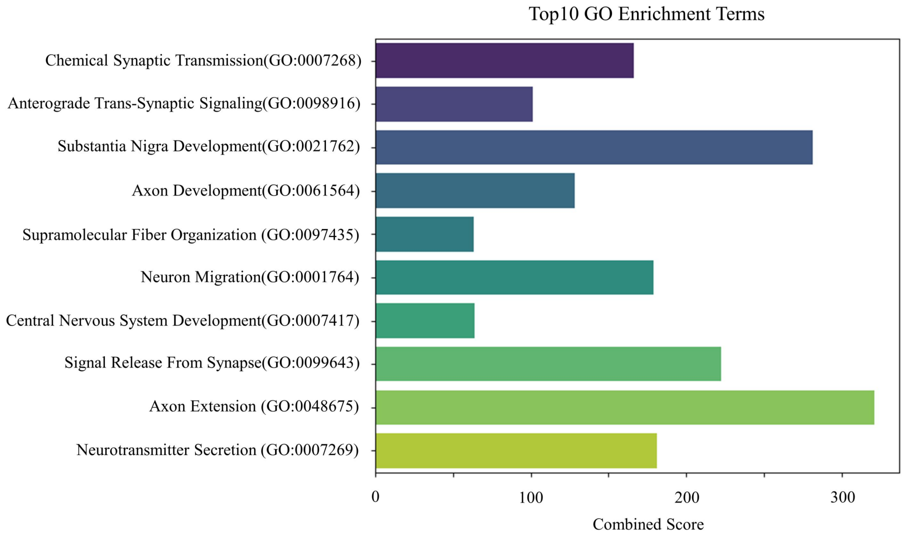

Figure S13. GO enrichment terms for differentially expressed genes identified on slice 151669. The bar chart utilizes an continuous color gradient (ranging from dark purple to lime green) to create an immediate visual data hierarchy, with colors mapped to the values of the Combined Score.

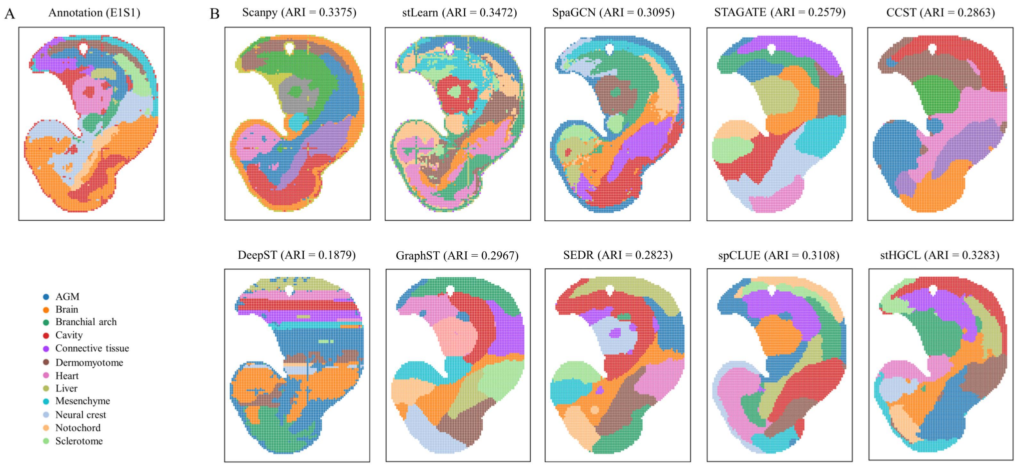

Figure S14. Visualization of different methods' clustering results on MOSTA slice E1S1. (A) Annotation structure on slice E1S1. (B) Domain identification on slice E1S1 by stHGCL and nine baseline methods.

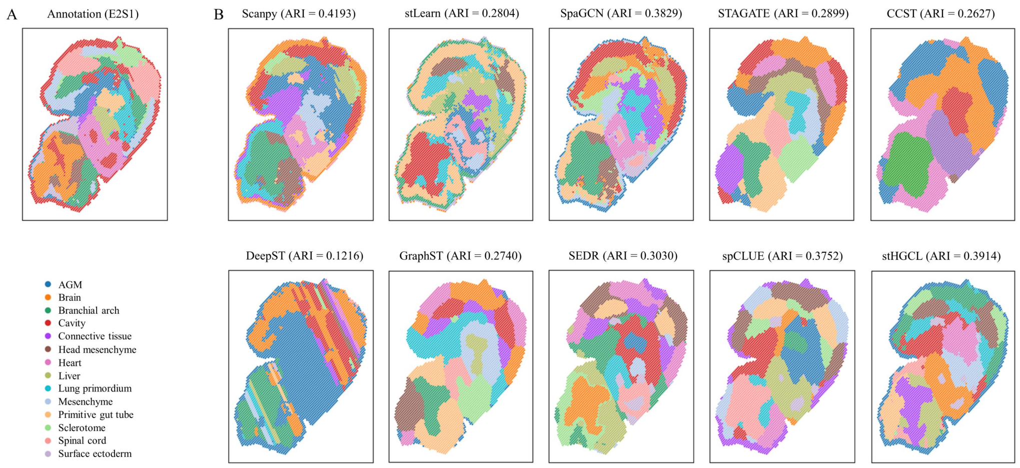

Figure S15. Visualization of different methods' clustering results on MOSTA slice E2S1. (A) Annotation structure on slice E2S1. (B) Domain identification on slice E2S1 by stHGCL and nine baseline methods.

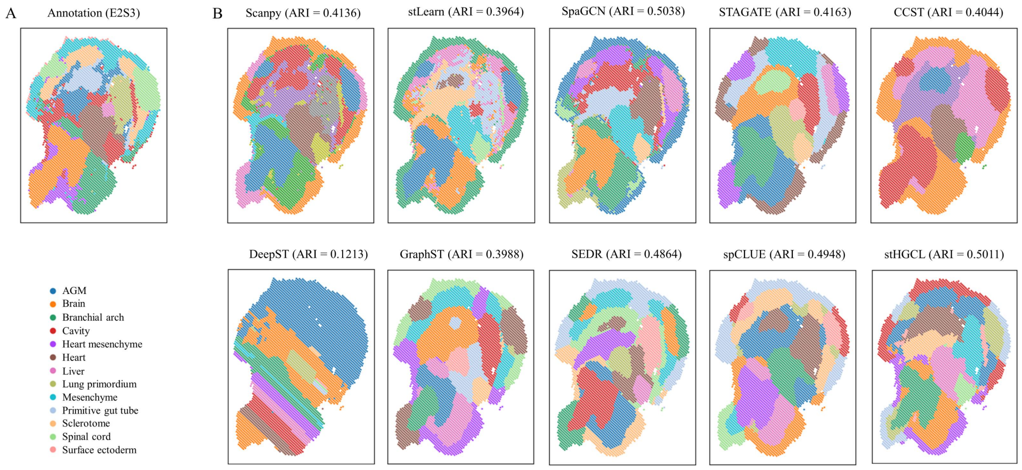

Figure S16. Visualization of different methods' clustering results on MOSTA slice E2S3. (A) Annotation structure on slice E2S3. (B) Domain identification on slice E2S3 by stHGCL and nine baseline methods.

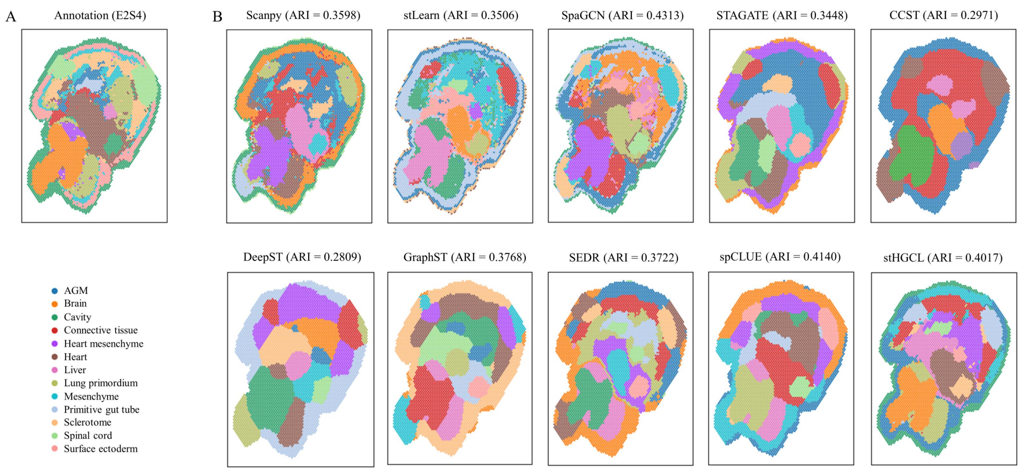

Figure S17. Visualization of different methods' clustering results on MOSTA slice E2S4. (A) Annotation structure on slice E2S4. (B) Domain identification on slice E2S4 by stHGCL and nine baseline methods.

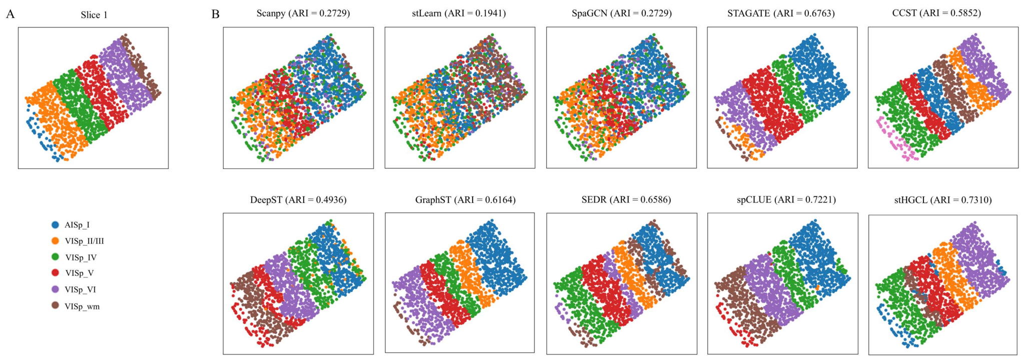

Figure S18. Visualization of different methods' clustering results on BARISTA slice 1. (A) Annotation structure on slice 1. (B) Domain identification on slice 1 by stHGCL and nine baseline methods.

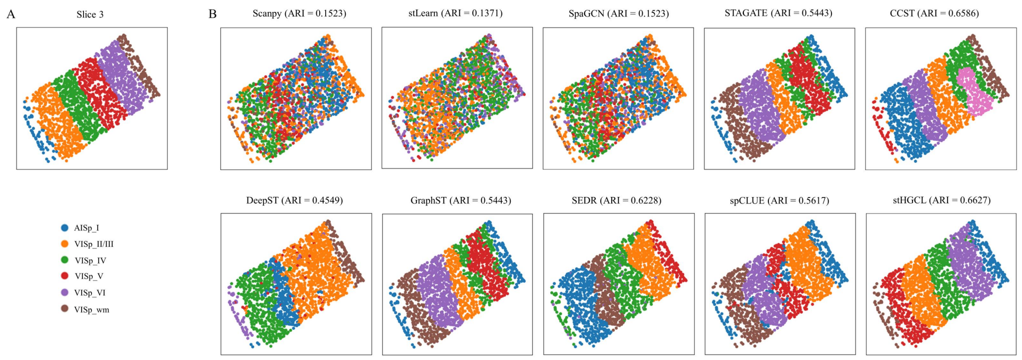

Figure S19. Visualization of different methods' clustering results on BARISTA slice 3. (A) Annotation structure on slice 3. (B) Domain identification on slice 3 by stHGCL and nine baseline methods.

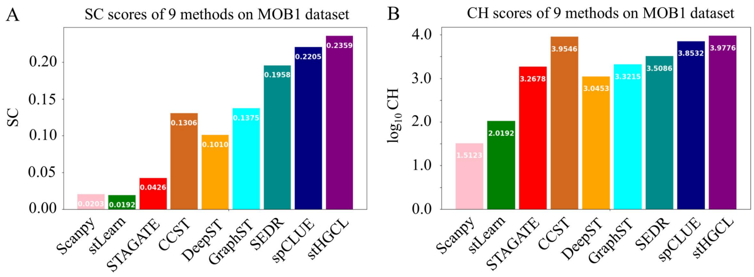

Figure S20. Comparison of SC and CH scores on the MOB1 dataset. (A) SC scores. (B) CH scores, CH scores were $\log_{10}$ transformed for presentation.

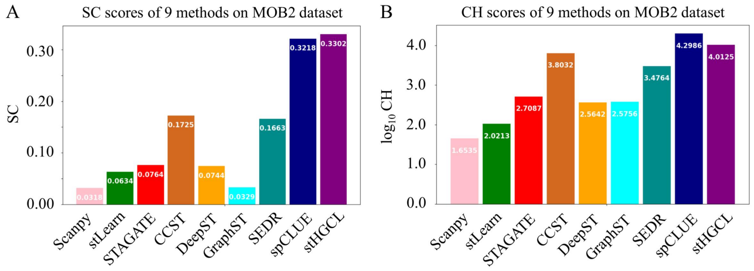

Figure S21. Comparison of SC and CH scores on the MOB2 dataset. (A) SC scores. (B) CH scores, CH scores were $\log_{10}$ transformed for presentation.

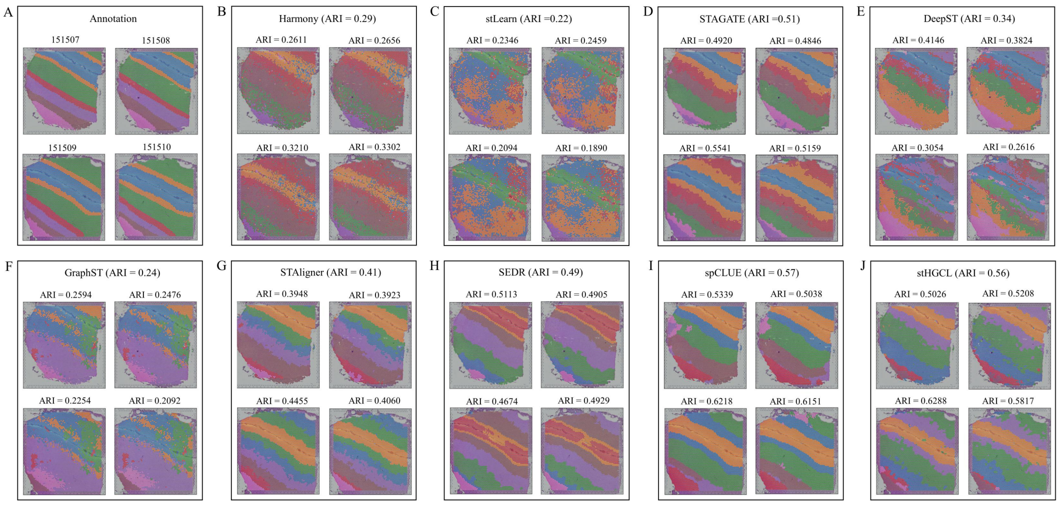

Figure S22. Multi-slice integration performance on DLPFC Sample 1. (A) Annotations for DLPFC Sample 1. (B–J) Spatial domain identification by (B) Harmony, (C) stLearn, (D) STAGATE, (E) DeepST, (F) GraphST, (G) STAligner, (H) SEDR, (I) spCLUE, and (J) stHGCL.

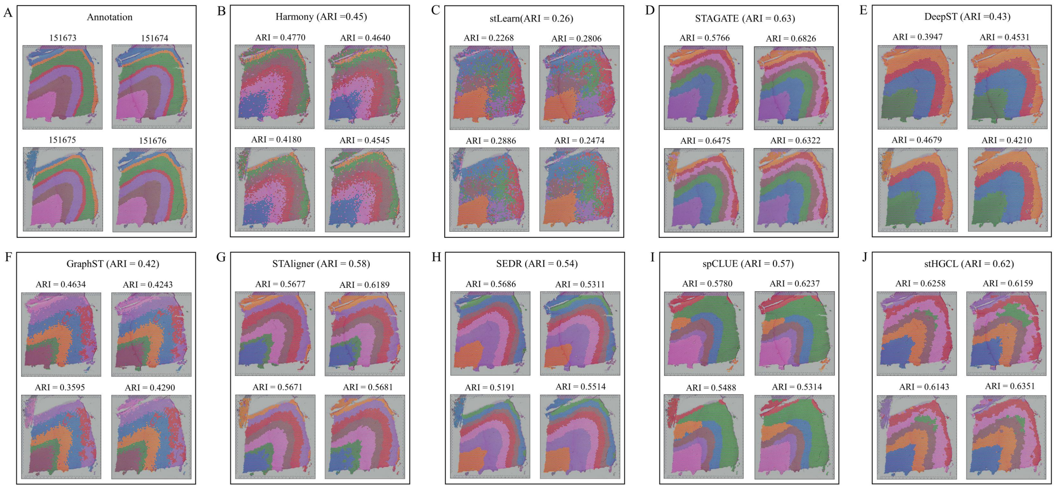

Figure S23. Multi-slice integration performance on DLPFC Sample 3. (A) Annotations for DLPFC Sample 3. (B–J) Spatial domain identification by (B) Harmony, (C) stLearn, (D) STAGATE, (E) DeepST, (F) GraphST, (G) STAligner, (H) SEDR, (I) spCLUE, and (J) stHGCL.

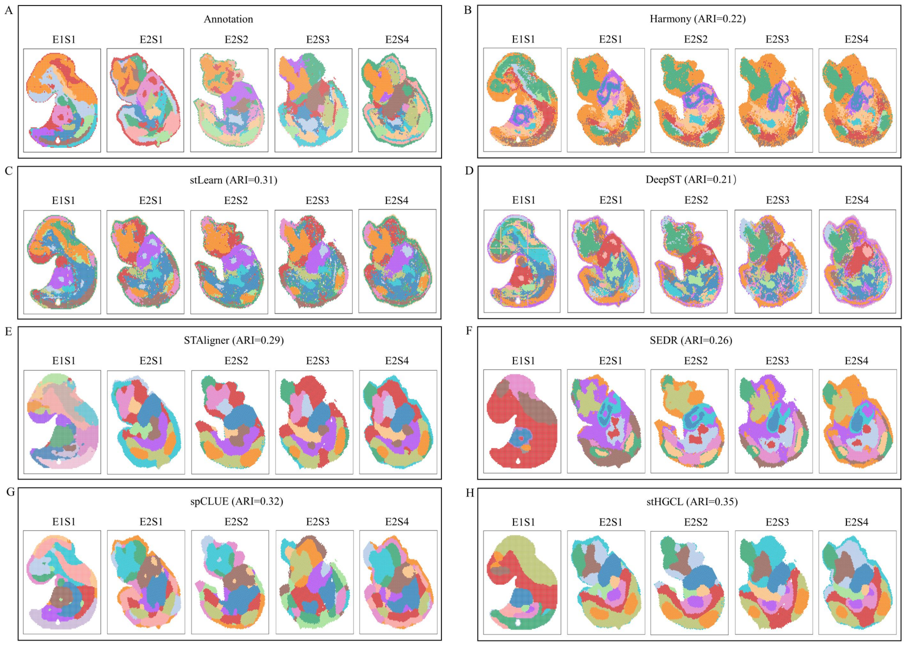

Figure S24. Multi-slice integration performance on MOSTA. (A) Annotations for MOSTA. (B–H) Spatial domain identification by (B) Harmony, (C) stLearn, (D) DeepST, (E) STAligner, (F) SEDR, (G) spCLUE, and (H) stHGCL.

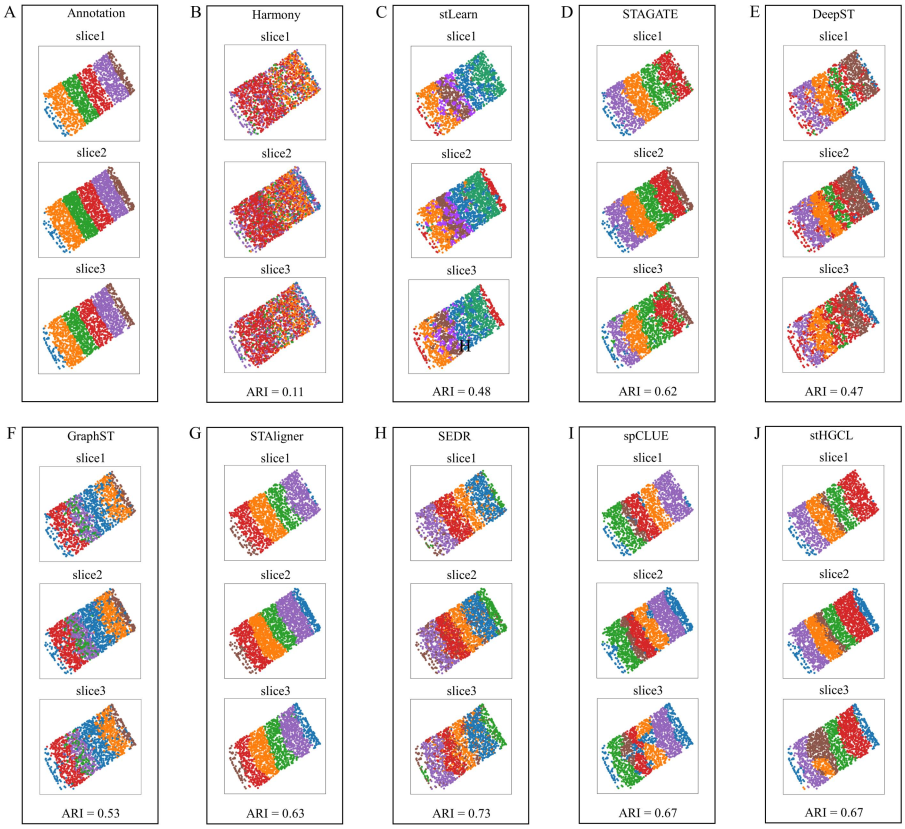

Figure S25. Multi-slice integration performance on BARISTA. (A) Annotations for BARISTA. (B–J) Spatial domain identification by (B) Harmony, (C) stLearn, (D) STAGATE, (E) DeepST, (F) GraphST, (G) STAligner, (H) SEDR, (I) spCLUE, and (J) stHGCL.

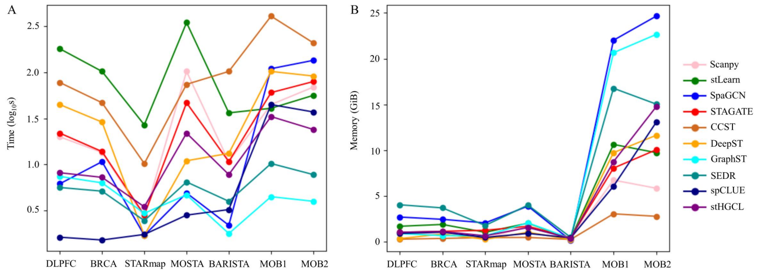

Figure S26. Comparison of running time and memory cost across single-slice datasets. For datasets that include multiple slices, the average time or memory usage across slices is presented. (A) Running time, running time was $\log_{10}$ transformed for presentation. (B) Memory cost.

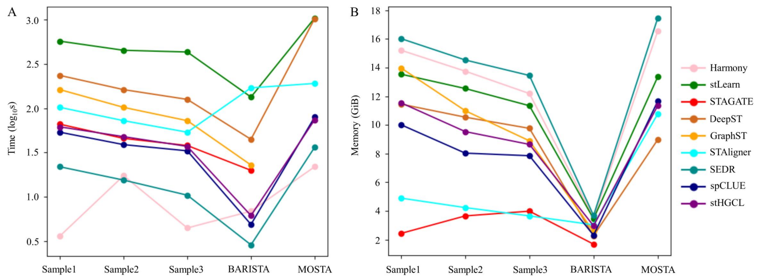

Figure S27. Comparison of running time and memory cost across multi-slice datasets. STAGATE, and GraphST were only evaluated on Sample1, Sample2, Sample3, and BARISTA datasets as they are not applicable to unaligned slices. (A) Running time, running time was log_{10} transformed for presentation. (B) Memory cost.

4. Supplementary Tables

Table S1: Comparison of spatial domain identification methods. In Table S1, “Model” indicates the key strategies employed by each method, “Spot embedding” indicates whether the method simultaneously learns latent spot embeddings and identifies spatial domains, “Single-slice” and “Multi-slice” indicate whether the method can be applied to single-slice or multi-slice data for identifying spatial domains, “Spatial information” indicates whether spatial coordinates were utilized in generating embeddings for clustering, and “Deep learning” indicates whether the method is built on a deep learning framework. The above features are summarized based on each method’s original publication.

Tabe S1. Summary of features of some methods for detecting spatial domains.

Method	Year	Model	Spot embedding	Single slice	Muiti slice	Spatial informatio	Deep learning
Scanpy	2018	Principle component analysis+louvain	√	√			
Harmony	2019	Maximum Diversity Clustering+Iterative Correction	√		√		
stLearn	2020	Spatial morphological gene expression normalization	√	√	√	√	√
SpaGCN	2021	Graph convolutional network		√		√	√
STAGATE	2022	Graph attention autoencoder	√	√	√	√	√
CCST	2022	DGI approach	√	√		√	√
DeepST	2022	Graph Neural Networks+Denoising Autoencoder+Multi-modal Fusion	√	√	√	√	√
GraphST	2023	Modified DGI approach	√	√	√	√	√
STAligner	2023	Graph attention autoencoder + mutual nearest neighborGraph	√		√	√	√
SEDR	2024	Variational graph autoencoder + masked self-supervised	√	√	√	√	√
spCLUE	2025	Multi-view graph network+Contrastive learning+Attention mechanism	√	√	√	√	√
stHGCL	2025	Spot-gene heterogeneous+Dual stage encoder+Contrastive learning	√	√	√	√	√

Table S2. Datasets used for method evaluation.

Dataset	Platform	Organism	Tissue	Slice	Spot	Gene
DLPFC	10x Visium	Human	Dorsolateral pre-frontal cortex	151507	4226	33538
				151508	4226	33538
				151509	4384	33538
				151510	4634	33538
				151669	3661	33538
				151670	3498	33538
				151671	4110	33538
				151672	4015	33538
				151673	3639	33538
				151674	3673	33538
				151675	3592	33538
				151676	3460	33538
BRCA	10x Visium	Human	Breast tumor	/	3798	36601
STARmap	STARmap	Mouse	Visual cortex	/	1207	1020
MOB1	Slide-seqV2	Mouse	Olfactory bulb	/	20139	11750
MOB2	Stereo-seq	Mouse	Olfactory bulb	/	19109	14376
MOSTA	Stereo-seq	Mouse	Embryo	E1S1	5913	25568
				E1S2	5292	23756
				E1S3	4356	24107
				E1S4	5059	24238
				E1S5	5797	23398
BARISTA	BaristaSeq	Mouse	Cortex	slice1	1525	79
				Slice2	2042	79
				Slice3	1690	79
